# Exploration of the Modulatory Property Mechanism of ELeng Capsule in the Treatment of Endometriosis Using Transcriptomics Combined With Systems Network Pharmacology

**DOI:** 10.3389/fphar.2021.674874

**Published:** 2021-06-18

**Authors:** Weilin Zheng, Jie Wang, Jiayi Wu, Tao Wang, Yangxue Huang, Xuefang Liang, Lixing Cao

**Affiliations:** ^1^ The Second Clinical College of Guangzhou University of Chinese Medicine, Guangzhou, China; ^2^ Department of Gynaecology, The Second Affiliated Hospital of Guangzhou University of Chinese Medicine, Guangzhou, China

**Keywords:** endometriosis, mRNA transcriptome analysis, network pharmacology, ELeng Capsule, traditional Chinese medicine

## Abstract

Endometriosis is a common gynecological disease and causes severe chronic pelvic pain and infertility. Growing evidence showed that traditional Chinese medicine (TCM) plays an active role in the treatment of endometriosis. ELeng Capsule (ELC) is a Chinese medicine formula used for the treatment of endometriosis for several years. However, the mechanisms of ELC have not been fully characterized. In this study, network pharmacology and mRNA transcriptome analysis were used to study various therapeutic targets in ELC. As a result, 40 compounds are identified, and 75 targets overlapped with endometriosis-related proteins. The mechanism of ELC for the treatment of endometriosis is based on the function modules of inducing apoptosis, inhibiting angiogenesis, and regulating immunity mainly through signaling molecules and interaction (neuroactive ligand–receptor interaction), immune system–associated pathways (toll-like receptor signaling pathway), vascular endothelial growth factor (VEGF) signaling, and MAPK signaling pathway based on network pharmacology. In addition, based on RNA-sequence analysis, we found that the mechanism of ELC was predominantly associated with the regulation of the function modules of actin and cytoskeleton, epithelial–mesenchymal transition (EMT), focal adhesion, and immunity-associated pathways. In conclusion, ELC exerted beneficial effects on endometriosis, and the potential mechanism could be realized through functional modules, such as inducing apoptosis and regulating angiogenesis, cytoskeleton, and EMT. This work not only provides insights into the therapeutic mechanism of TCM for treating endometriosis but also offers an efficient way for drug discovery and development from herbal medicine.

## Introduction

Endometriosis is a common gynecological disease and causes severe chronic pelvic pain and infertility, which affect the physical and mental health and quality of life of women. The pathogenesis of endometriosis has not been fully elucidated; an increasing body of research shows that it is associated with inflammation, immunity, angiogenesis, and epithelial–mesenchymal transition (EMT) (Khan et al., 2012; Morotti et al., 2017).

Currently, the treatment of endometriosis is mainly based on surgery and pharmacological treatment (Dunselman et al., 2014). Though beneficial, conventional treatments of endometriosis have significant limitations. In recent years, traditional Chinese medicine (TCM) plays an active role in the treatment of endometriosis such as dysmenorrhea, chronic pelvic pain, abnormal uterine bleeding, and infertility by regulating inflammation, immunity, and angiogenesis (Flower et al., 2012; Dunselman et al., 2014). Blood stasis syndrome in TCM is considered appearing in endometriosis. The Chinese preparation ELeng Capsule (ELC) is one of the famous *Huoxue Huayu* prescriptions and is currently used as an in-hospital preparation in the Guangdong Provincial Hospital of Chinese Medicine to relieve the symptoms of endometriosis-associated pain and dysmenorrhea for nearly 20 years. ELC is an empirical formula of Chinese herbs created by Yi Situ, a famous expert in Chinese medicine in Guangdong. The clinical practice and animal experiments have suggested that ELC could reduce dysmenorrhea and endometriosis-associated pain through inhibiting adhesion and inflammation and regulating immunity (Huang and Jiang, 2008; Xu et al., 2010). However, the mechanisms of action of ELC have not been fully characterized.

Chinese medicine compounds exist in complex mixtures and may contain thousands of compounds. Therefore, it is difficult to explain the principle of compatibility of Chinese medicine ingredients and analyze relevant results. Network pharmacology can predict the profiles of targets and pharmacological actions of herbal compounds to reveal “compounds/drugs-genes/targets-disease,” which will improve current drug discovery strategies (Mihalyi et al., 2006; Rogers et al., 2009; Zeng et al., 2017). In addition, the development of multi-omics technology also provides new tools for research on TCM. The high-throughput RNA-sequencing (RNA-seq) has been used to reveal molecular mechanisms and explore biomarkers for diagnosis and treatment (Duan et al., 2018; Zhang et al., 2019). These new methods could provide the basis for clarifying the therapeutic mechanisms of herbal medicine.

In this study, RNA-sequencing combined with network pharmacology was performed to identify targets regulated by ELC treatment. Then, autologous transplantation of the endometriosis rat model was used to evaluate the *in vivo* effect of ELC on endometriosis. We aim to provide a reliable way for subsequent experimental verification and new drug research and development.

## Materials and Methods

### Workflow of Network Pharmacology Combined With RNA-Sequence Approach

**FIGURE 1:**
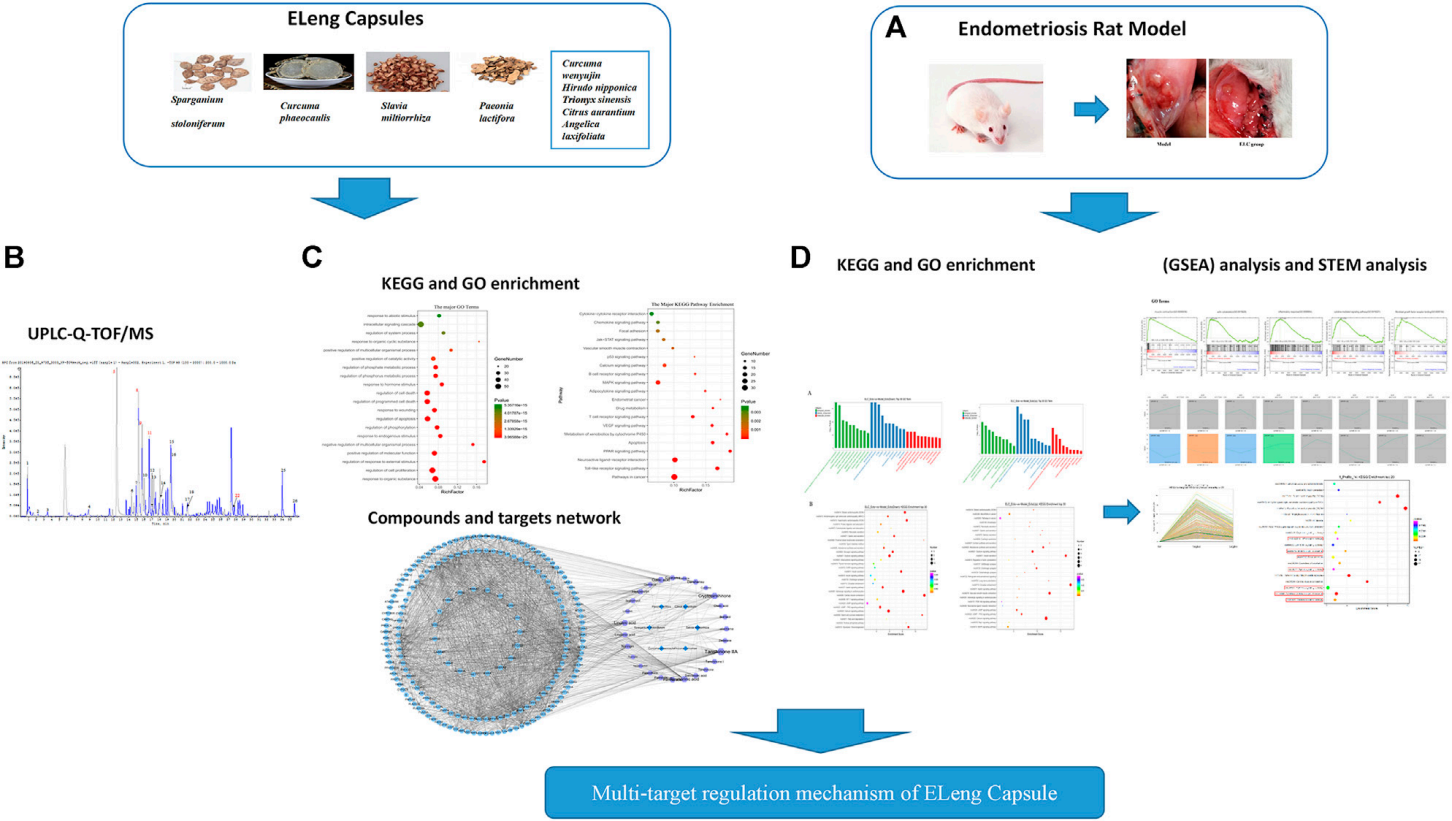
Workflow of network pharmacology combined with the RNA-sequence approach. **(A)** Endometriosis model rats were established and used to verify the core targets. **(B)** The compounds of ELCs were identified by ultra-performance liquid chromatography/quadrupole time-of-flight mass spectrometry (UPLC-Q-TOF/MS). **(C)** Network pharmacology was used to analyze the compounds-targets network of ELC. **(D)** RNA-sequencing was used to identify differentially expressed genes (DEGs). Biological functions and pathways were determined through gene ontology and Kyoto Encyclopedia of Genes and Genomes (KEGG) pathway analyses. Gene set enrichment analysis (GSEA) and STEM analysis were used to further analyze the genetic network and modular genetics.

The workflow is shown in Figure 1: (A) Endometriosis model rats were established and used to verify the core targets. (B) The compounds of ELCs were identified by ultra-performance liquid chromatography/quadrupole time-of-flight mass spectrometry (UPLC-Q-TOF/MS). (C) Network pharmacology was used to analyze the compounds-targets network of ELC. (D) RNA-sequencing was used to identify differentially expressed genes (DEGs). Biological functions and pathways were determined through gene ontology and Kyoto Encyclopedia of Genes and Genomes (KEGG) pathway analyses. Gene set enrichment analysis (GSEA) and STEM analysis were used to further analyze the genetic network and modular genetics.

### The Preparation of ELeng Capsule

ELC was provided by the pharmaceutical department of The Second Affiliated Hospital of Guangzhou University of Chinese Medicine (Guangdong Provincial Hospital of Chinese Medicine), Guangzhou, China. The validated information of herb/plant names with taxonomic validation was collected from The Plant List (http://www.theplantlist.org). ELC composed of E’zhu (*Curcuma phaeocaulis* Valeton), Sanleng [*Sparganium stoloniferum* (Buch.-Ham. ex Graebn.) Buch.-Ham. ex Juz. (Typhaceae)], Danshen [*Salvia miltiorrhiza* Bunge (Lamiaceae)], Chishao (*Paeonia lactiflora* Pall.), Shuizhi (*Hirudo nipponia*), Biejia (*Trionyx sinensis*), Zhike (*Citrus aurantium*), and Danggui [*Angelica sinensis* (Oliv.) Diels] (Table 1). The weight ratio of *C. phaeocaulis* (E’zhu), *S. stoloniferum* (Sanleng), *S. miltiorrhiza* (Danshen), *Hirudo nipponia* (Shuizhi), *Trionyx sinensis* (Biejia), *P. lactiflora* (Chishao), *C. aurantium* (Zhike), and *A. sinensis* (Danggui) is 2: 2: 5: 1: 5: 5: 4: 3.4. Each capsule weighed 0.45 g, which is equal to 1.61 g of crude drug. According to the extraction method of volatile oil recorded in the Chinese Pharmacopoeia, the volatile oil was extracted by steam distillation. The water extraction and alcohol precipitation solution was filtered and spray-dried to obtain a dry extract powder.

**TABLE 1:** The major herbs of ELeng Capsule.

Herb	Component	Effect
*Curcuma phaeocaulis* Valeton (E’zhu)	Ginger plant, Wen Yujin Curcuma Wenyujin Y.H. Chen et C. Ling, rhizome	Treatment of mass in the abdomen, amenorrhea due to blood stasis, distension, and pain due to stagnation of undigested food
*Sparganium stoloniferum* (Buch.-Ham. ex Graebn.) Buch.-Ham. ex Juz. (Typhaceae) (Sanleng)	Black-triangular plant, *Sparganium stoloniferum* Buch.-Ham, tubers	To break blood, move qi, relieve pain, and disperse accumulation
*Salvia miltiorrhiza* Bunge (Lamiaceae) (Danshen)	*Salvia miltiorrhiza* Bunge (Lamiaceae), dry roots, and rhizomes	To quicken blood and dispel stasis, regulate menstruation and relieve pain, nourish blood and calm spirit, cool blood and disperse swelling abscess
*Hirudo nipponia* (Shuizhi)	Mink animal otter, *Hirudo nipponia* Whitman, dry body	To clear heat and resolve toxin, disperse swelling and relieve pain
*Trionyx sinensis* (Biejia)	Cyprinidae, *Trionyx sinensis* Wiegmann, carapace	Nourish the Yin and suppress Yang, dispel stasis and dissipate knots, soften hardness
*Paeonia lactiflora* Pall.(Chishao)	Ranunculaceae, peony, *Paeonia lactiflora* pall, root	Treatment of maculation in epidemic diseases, spitting of blood, epistaxis, inflammation of the eye, pain in the chest and lateral thorax, amenorrhea, dysmenorrhea, mass formation in the abdomen, traumatic injuries
*Citrus aurantium* L.(Zhike)	*Citrus aurantium* L., a dried, immature fruit of the cultivar	Clear heat and activate blood, promoting circulation of qi and blood
*Angelica sinensis* (Oliv.) Diels (Danggui)	*Angelica sinensis* (Oliv.) Diels, root	To nourish blood and regulate menstruation, quicken blood, relieve pain, moisten intestines, and relieve constipation

The original medicinal materials were purchased from Guangdong KangMei Pharmaceutical Co., Ltd. The quality of the raw herbs was controlled according to the Pharmacopoeia of the People’s Republic of China (2020). The patent certificate number is 432493. ELC was obtained with the water extraction–alcohol precipitation method. The validated information of major herbs, including the location, used part, family, and genus, is shown in Supplementary Table S1.

### UPLC-Q-TOF/MS Analysis

For the preparation of compounds, 20 ml of 50% ethanol solution was added to 1 g of medicinal powder of ELC. The mixture was ultrasonicated for 30 min and centrifuged at 1.2 × 10^4^ rpm for 5 min, and the supernatant was removed. The chromatographic conditions were as follows: The UPLC device was Agilent 1290 UPLC, and the column was Agilent SB-C18, 2.1 × 100 mm, 1.8 μm. The column temperature was 30°C, and the injection volume was 5 μl. The detection wavelength was 254 nm. Phase A is 0.1% formic acid aqueous solution, and phase B is acetonitrile. Gas chromatography-mass spectrometry (GC-MS) was performed using Agilent 7890A/5975C. The column was Agilent HP-5MS, 30 m × 250 μm × 0.25 μm. The inlet temperature was 250°C, ion source temperature was 230°C, and quadrupole temperature was 150°C. The compounds were tentatively characterized based on their retention time, mass accuracy of precursor ions, MS/MS spectra, and fragmentation pathways, referring to the SCIEX natural product HR-MS/MS Spectral Library and previous literatures. The conditions of UPLC-Q-TOF/MS are listed in Supplementary Table S2. Methanol and acetonitrile of chromatographic grade were supplied by Merck Chemicals (Shanghai, China). 2-Chloro-L-phenylalanine was bought from Hengbai Biotechnology (Shanghai, China).

### Network Pharmacology Analysis of ELeng Capsule

#### Candidate Compound Database

Compounds of ELC were compiled from the STITCH database (http://stitch.embl.de/), the traditional Chinese medicine systems pharmacology (TCMSP) database (http://tcmspw.com/tcmsp.php), and the Universal Natural Products Database (UNPD) (Gu et al., 2013). The structures of compounds were retrieved from the PubChem dataset (https://www.ncbi.nlm.nih.gov/pccompound/). All three-dimensional molecular structures of active ingredients were obtained from PubChem in mol2 format.

#### Candidate Endometriosis-Associated Genes

The genes of endometriosis of patients were collected from GeneCards (https://www.genecards.org/), Online Mendelian Inheritance in Man (OMIM) (https://www.omim.org/), and GenBank (https://ncbiinsights.ncbi.nlm.nih.gov/tag/genbank/) databases. The targets/proteins were researched in the UniProt database (https://www.uniprot.org). The three-dimensional structures of proteins related to endometriosis were obtained from the Research Collaboratory for Structural Bioinformatics Protein Data Bank (PDB) (www.rcsb.org/pdb/home/home.do).

#### GO and KEGG Enrichment Analyses

Furthermore, we performed GO enrichment and KEGG pathway enrichment analyses of ELC-associated targets. We used the web-based search engine, DAVID, to determine over-represented GO terms and KEGG pathways with thresholds of an enrichment score >2, count >5, and *p* < 0.05.

#### Network Construction

The online search tool for recurring instances of neighboring genes (STRING, version 9.1) (http://string-db.org/) was used to predict the protein–protein interactions. The compounds-targets networks were constructed using Cytoscape software 3.7.2 (http://cytoscape.org/). The related parameters were calculated to detect significant nodes (Shannon et al., 2003).

### Animal Model Establishment and Treatments

This study used female Sprague Dawley (SD) rats (age: 8 ± 1 weeks, weight: 220–230 g). The rats are from the Experimental Animal Center of Guangdong Province (Guangzhou, Guangdong, China), and the certificate number is 44007200054328. The rats were housed at 20 ± 2°C on a 12-h light/dark cycle, with *ad libitum* access to food and water, and raised in the Laboratory Animal Center of The Second Clinical Medical College of Guangzhou University of Chinese Medicine (Guangzhou, Guangdong, China). The rats were housed five per cage. The animals and the protocols were approved by the Guangdong Provincial Hospital of Chinese Medicine Committee on the Use of Live Animals for Teaching and Research (No. SZY2016007). And disposal methods were in accordance with animal ethics standards.

### Surgical Operation

A model of endometriosis was established through allotransplantation in rats. All operational procedures were conducted under sterile conditions. The rats were anesthetized with 3% pentobarbital sodium prior to performing a vertical incision in the abdomen. The right uterus of each rat was removed and immediately placed in a saline solution. Briefly under sterile conditions, the endometria were separated from the myometria and cut into 0.5 × 0.5 cm pieces. The endometria were sutured onto the peritoneum close to blood vessels in each abdominal wall using a 5-0 absorbable suture. After transplantation (28 days), the growth of the ectopic endometrium was observed via gross and microscopic examination. The endometriosis rat models established successful are following criteria that endometrial explants developed into ovoid, large, fluid-filled, well-vascularized, and cystic lesions (Vernon and Wilson, 1985). The volume of the ectopic endometrium was detected by a vernier caliper with the volume formula (length × width × height × 0.52).

After 28 days of auto-transplantation, endometriosis models were successfully established. The 40 endometriosis SD rats were randomly divided into four groups: the ELC low-dose group (0.5 g/kg/d of ELC), the ELC middle-dose group (1 g/kg/d of ELC), the ELC high-dose group (2 g/kg/d of ELC), and the model group (10 ml/kg/d of 0.9% sodium chloride). The middle-dose group was equivalent to the clinical dose. For animal experiments, the interior powder (0.45 g/capsule) after removed from the shells of ELC was blended with appropriate saline as a working mixture for use. Rats were fed by gavage once a day for 28 days.

In addition, another ten SD rats were selected as the control group and fed routinely. At the end of ELC treatment, the eutopic endometrium from the control group and ectopic endometrium from the endometriosis model rats were collected. The volumes of ectopic endometrial lesions in each group before (V1) and after (V2) treatment were measured. The tissues were used for histopathology analysis, immunohistochemistry, RNA-sequencing, and quantitative polymerase chain reaction (qPCR) validation.

### Hematoxylin and Eosin and Masson’s Trichrome Staining

Sections from different groups were stained with hematoxylin and eosin (HE). And Masson’s trichrome staining was used for the detection of collagen fibers in tissues. The stained areas of the sections were observed under an optical microscope (Nikon, Japan) and NIS-Elements. Fibrosis analysis was performed using the ImageJ software to analyze the proportion of blue staining.

### Transmission Electron Microscopy Analysis

The tissue samples were fixed immediately with 1% glutaraldehyde and 4% formalin for 6 h at 4°C and rinsed in 0.1 M cacodylate buffer overnight. Ultrathin sections were prepared with Ultratome Nova, double-stained with uranyl acetate and lead citrate, and examined under an electron microscope.

### Terminal Deoxynucleotidyl Transferase–Mediated Digoxigenin-dUTP Nick-End Labeling Assay

Apoptosis was detected using the terminal deoxynucleotidyl transferase biotin-dUTP nick-end labeling (TUNEL) apoptosis detection kit (C1086, Beyotime Biotechnology, China) according to the manufacturer’s instructions (n = 4 each group). The labeled apoptotic cells expressed green fluorescence under fluorescence microscopy. The Image J software was used for assessing the ratio.

### Monoclonal Antibody and Microvessel Density

Vascular endothelial cells were labeled with a CD34 monoclonal antibody, and the microvessel density (MVD) was counted (*n* = 6 each group). The dilution ratio of anti-CD34 antibody (1:500, ab185732, Abcam, United States) was used. Three dense microvessel areas were selected for each slice, and the microvessels were counted by a double-blind method under high power (200×).

### Immunohistochemical Staining

The sections were stained by IHC staining to detect the expression of factors in the VEGF family and *α*-SMA. After the antigen was repaired, primary antibodies were added at 4°C overnight, and then secondary antibodies were added at room temperature for 1 h, avoiding light. Diaminobenzidine (DAB) was used for staining, and neutral gum was used to seal pieces. The antibodies were anti-VEGFA (1:1000, ab81289, Abcam, United States), anti-VEGFB (1:1000, ab81289, Abcam, United States), anti-VEGFC (1:1000, ab81289, Abcam, United States), and *α*-SMA (1:1000, 14395-1-AP, Proteintech, United States) (*n* = 4 each group). The Image J software was used for assessing the mean optical density.

### ELISA

The serum of abdominal aorta was prepared for analysis. Thereafter, the samples and standard samples were diluted with distilled water and applied to ELISA plates. The VEGFA (*Cloud-Clone Corp*, Wuhan, China) and VEGFB (*Cloud-Clone Corp*, Wuhan, China) concentrations were determined according to the manufacturer’s instructions. Absorbance levels were measured at 450 nm using an ELISA reader.

### RNA-Sequencing of ELeng Capsule

#### The Design of RNA-Sequencing Analysis

The model rats of the 1 mg/kg/d dose ELC group were chosen for further biological experiment because of the dose equivalent to the human dose. We randomly selected transcriptomes from eutopic endometrium tissues and ectopic endometrium tissues for analyses in the control, model, and ELC groups. Sample groups consisted of *n* = 4 eutopic endometria, including (Con_Euto), (Model_Euto), and (ELC_Euto) groups, *n* = 4 model group ectopic endometriotic lesions (Model_Ecto), and *n* = 5 ELC group ectopic endometriotic lesions (ELC_Ecto). A crossover comparison was performed in the following three paired groups to identify genes that were differentially regulated in the model group and ELC group: Con_Euto *vs.* Model_Ecto groups; Con_Euto *vs.* ELC_Ecto groups; and Model_Ecto *vs.* ELC_Ecto groups.

#### Gene Ontology Terms and Kyoto Encyclopedia of Genes and Genomes Analyses

Preparation of transcriptome libraries and sequencing were performed by Shanghai OE Biotech Co. (Shanghai, China). Raw data (raw reads) were processed using Trimmomatic (Bolger et al., 2014). Multiple hypothesis testing correction for the treatment effect was performed using the false discovery rate (FDR) method. GO and KEGG enrichment analyses of differentially expressed genes (DEGs) were, respectively, performed using R studio. The GO analysis provides three structured networks of defined terms to describe gene product attributes: cellular compartment (CC), biological process (BP), and molecular function (MF). Pathway analysis was applied to determine the significant pathways of DEGs according to KEGG, MapSplice, and Reactome Functional Interaction network and external interaction databases (Reactome database). Fisher’s exact test was used to identify significantly enriched pathways, and the threshold of significance was defined as *p <* 0.05 and FDR <0.05.

#### Gene Set Enrichment Analysis

In this study, 1,000 genes of permutations were set to generate a null distribution for the enrichment score in the hallmark gene sets and functional annotation gene sets. The publicly available GSEA software package (www.broad.mit.edu) was used for leading edge analysis to examine genome-wide expression profiles (Subramanian et al., 2005). Nominal *p <* 0.05, FDR <0.25, and gene set size >100 were defined as the cut-off criteria. The aim of this analysis was to determine whether the members of the identified gene ontology and KEGG pathways were randomly distributed throughout the ranked gene list or concentrated at the top or bottom.

#### Trend Modular Analysis (STEM Analysis)

Short Time-series Expression Miner (STEM) is a software program, which could be designed for the analysis of short time-series microarray gene expression data (Ernst and Bar-Joseph, 2006). This approach was used to identify the profiles of the “up to down” model from Con_Euto to Model_Ecto to ELC_Ecto. The results of STEM analysis could help discover the regulation mechanism of ELC.

#### Construction of the Protein–Protein Interaction Network

The STRING database provides comprehensive information regarding interactions between proteins. Subsequently, the PPI network was visualized using Cytoscape (version 3.7.2; National Institute of General Medical Sciences, Bethesda, MD, United States) (Shannon et al., 2003). The PPI network was used to filter modules based on the Molecular Complex Detection (MCODE) plugin in Cytoscape with the following conditions: degree cut-off = 2; k-core = 2; node score cut-off = 0.2; and max depth = 100.

#### Quantitative Reverse Transcription-PCR

qRT-PCR was performed to validate the gene expression data obtained from deep sequencing. Total mRNA was extracted using the TRIzol reagent (Invitrogen, Carlsbad, CA) according to the instructions provided by the manufacturer. The first strand of cDNA was synthesized using primers designed in our laboratory. The RT product was amplified using SYBR Green on a 7500 Real-Time PCR System (Thermo Fisher Scientific Inc., Waltham, MA, United States). All samples were run in triplicate, and the relative gene expression was analyzed according to the 2^−ΔΔCt^ method. The sequencing accessions of the primers were myogenin (Myog), SET and MYND domain containing 1 (Smyd1), SIX homeobox 1 (Six1), calcium voltage-gated channel subunit alpha1 S (Cacna1s), eukaryotic translation elongation factor 1 alpha 2 (Eef1a2), ryanodine receptor 1 (Ryr1), actinin alpha 2 (Actn2), myogenic differentiation 1 (Myod1), mitogen-activated protein kinase 12 (Mapk12), and myosin heavy chain 4 (Myh4). Gene expression levels were normalized to that of ACTB. The primer sequences are shown in Table 2.

**TABLE 2:** Primer sequences used for real-time PCRs.

No.	Gene symbol	Forward primer	Reverse primer
1	Myog	CGACCTGATGGAGCTGTA	GGTGGACAGGAAGGTAGT
2	Smyd1	ACC​GTC​TAT​TTA​ACA​AGG​AAG​C	GCACCGTGGCATTTACTA
3	Six1	ATT​AGT​GAG​GGA​AAC​AAG​TGC	GTT​TGT​TGC​GTT​ACT​AAC​ATC​G
4	Cacna1s	CAC​CTG​GTT​CAC​CAA​CTT​TAT	CTG​ATT​CCT​CAT​GGA​GTC​G
5	Eef1a2	CCA​GCA​AAT​ACC​CTC​AAC​C	GTC​TTC​TCC​TTG​CCC​ATT​C
6	Ryr1	AGCCGTATGTACCTGAGT	GTG​GCG​TCT​TCC​TGT​AAT​C
7	Actn2	CCA​GCG​CCA​TGA​ATC​AGA​TA	CTC​CTC​CTG​GAT​CAT​GTA​CTC
8	Myod1	GACAGCAGGTGTGCATTC	TAG​TAG​CTC​CAT​GTC​CCA​GT
9	Mapk12	CAG​TGG​ACA​TTT​GGT​CTG​TTG	TGGTCCAGGTGGTCATTG
10	Myh4	CAAGGTGAAGAACGCCTA	TCCAGCTCGTGGATATGC
11	ACTB	GCG​AGT​ACA​ACC​TTC​TTG​C	TAT​CGT​CAT​CCA​TGG​CGA​AC

### Statistical Analysis

Data were analyzed using the Prism software (version 7.0; GraphPad Prism, San Diego, California, United States). All experimental data are presented as the mean ± standard error of the mean. The qPCR data were analyzed using two-tailed Student’s *t*-test. Unless otherwise indicated, *p <* 0.05 denotes statistical significance.

## Results

### UPLC-Q-TOF/MS Results for ELC

In this study, the compounds of ELC were identified by UPLC-Q-TOF/MS. According to the UPLC-Q-TOF/MS combined with the data obtained from the literature and databases, another 26 compounds were identified in the ELC compound preparation. Representative fingerprint chromatograms of ELC are displayed in Supplementary Figures S1–S3. The identified compounds of ELC are shown in Supplementary Table S3. In our previous study, a total of 14 compounds were identified based on GC-MS, namely, eucalyptol, D-camphor, isoborneol, L(−)-borneol, α-terpineol, β-elemene, γ-elemene, α-humulene, germacra, curcumenol, β-cyclocostunolide, curcumenone, pulmonary zederone, and ent-kaurene (Supplementary Figure S4 and Supplementary Table S4). Combined with our previous study results, we have identified a total of 40 compounds in ELC.

### Network Pharmacology Analysis

#### The Compounds’ Associated Targets of ELeng Capsule

Furthermore, based on the data obtained from the network pharmacology–related databases, we identified 27 potential compounds with 194 potential targets based on STITCH, TCMSP, and UNPD datasets. The result showed that these major targets were involved in angiogenesis, inflammation, immunity, cell adhesion, cell invasion, and other modules. Supplementary Table S5 shows the major compounds and targets of ELC. Combined with the target and previous research evidence, the results implied that tanshinone IIA, cryptotanshinone, rosmarinic acid, danshensu, tanshinone I, paeoniflorin, gallic acid, linoleic acid, *γ*-elemene, hesperetin, palmitic acid, naringin, *etc*., may be compounds that play the major role in endometriosis (Table 3).

**TABLE 3:** Mechanism of the compounds with potential therapeutic properties.

Herb name	Compound name	Molecular formula	Potential targets based on network pharmacology	Major mechanism	References
*Salvia miltiorrhiza*	Salvianolic acid A	C26H22O10	AKT1,BCL2,CDKN3,EIF3L,F10,PRSS1,CASP3,COL7A1,F7,PTPN6,CCND1	Anti-thrombosis; anti-fibrosis	Xu et al. (2018)
	Tanshinone IIA	C19H18O3	ACHE,ADRA1A,ADRB1,ADRB2,CASP3,CHRM1,F2,OPRM1,CHRM2,DPP4,RXRA,PTGS2,CHRM5,CHRNA7,OPRD1,CHRM3,CHRM4,DRD1,NFKB3,CYP1A1,EDN1,BCL2,FOS,TP53,CYP1A2,CYP3A4,ITGB3,JUN,MMP9	Reduce the expression of AGT, REN, ACE, ANGII, and AT2 in DRG neurons; reduce the VEGF/VEGFR2 pathway and CD146	Qi Zhang et al. (2016); Luo et al. (2021)
	Cryptotanshinone	C19H20O3	ADRA1A,ADRA1B,ADRA1D,ADRB1,ADRB2,APP,BCL2L1,BIRC5,CHRM1,CHRM3,CHRM4,CHRNA7,PTGS1,DRD1,CHRM5,PTGS2,CA2,OPRD1,CHRM2,TOP2A,OPRM1,NCOA2,PGR,GABRA1,NFKB3,STAT1,CCND1,TNF,EDN1	Anti-tumor, anti-inflammatory, neuroprotective, cardioprotective, visceral protective, anti-metabolic disorders; anti-tumor effects; STAT3-related pathways	Wu et al. (2020)
	Rosmarinic acid	C18H16O8	F2,ESR1,AR,PPARG,PTGS2,DPP4,PRSS1,NFKB3,IKBKB,CDKN3,EIF3L,MAPK1,CASP3,STAT1,CCL13,MGAM,IL2,NFATC3,CCND3,IL4R,IL5,CCL3,CD80,CD86,CCL11,CCR6,IDO1,SNCA,IGHG1,C3,C5	Anti-cancer properties; inhibit the proliferation of primary HESCs and T-HESCs	Yesil-Celiktas et al. (2010); Ferella et al. (2018)
	Salvianic acid A	C9H10O5	ACHE,ACTB,ADRA1D,ADRA2A,ADRA2B,ADRA2C,ADRB1,ADRB2,COL1A1,COL3A1,F2,TGFB1,HMOX1,PLAU		
	Tanshinone I	C18H12O3	F2,AR,PTGS2,RXRA,DPP4,HSP90AB1,PIK3CG,PRSS1,VEGFA,ICAM1,VCAM1		
	Linolenic acid	C18H30O2	PTGS1,PTGS2,NCOA2,O3FAR1,FADS2,FADS1,PPARA,PPARG,CD36,PLA2G6,PLA2G2A,PLA2G1B,PNPLA8		
*Paeonia lactiflora* Pall. (Chishao)	Paeoniflorin	C23H28O11	TNF,IL6,CD14,LBP,TLR4,HSF1,IL8	Relieve pain; anti-inflammatory through inhibiting TLR4/MMP-9/2/IL-1β signaling pathway	Fan et al. (2018); Chen et al. (2020)
	Paeoniflorigenone	C17H18O6	GABRA1	Induce apoptosis; suppress proliferation	Huang et al. (2017)
	γ-Elemene	C15H24	CHRM2,PTGS1,PTGS2,RXRA,ADRA1A,RXRA,GABRA2,GABRA1,GABRA6,PTGS1,CHRM3	Analgesic effects; anti-tumor	Chen et al. (2021)
*Citrus aurantium* L. (Zhike)	Neoeriocitrin	C27H32O15	TOP2A	Induce apoptosis; regulation of the MAPK and Akt signal transduction pathways	Park et al. (2017)
	Narirutin	C27H32O14	TOP2A	Cell signal transduction pathways in cancer	Memariani et al. (2020)
	Naringin	C27H32O14	TOP2A,CDKN3,TNF,RASGRF1,RAF1,PPARA,DPP4,PPARG,AKT1,NQO1,MMP9,BMP2,CCK,GHSR,IL8	Anti-tumor through cell signal transduction pathways in cancer (JAK–STAT pathway, PI3-kinase/Akt/mTOR signaling pathway, Notch pathway, NF-κB and cox-2 pathway, Wnt pathway, MAPK-ERK pathway, TGF-β signaling pathway); regulate angiogenesis	Memariani et al. (2020)
	Hesperetin	C16H14O6	PTGS1,ADRB1,PTGS2,HSP90AB1,PIK3CG,PRKACA,NCOA2,CAMTA3,CYP71B35,TAG1,AT4G35090,CAT1,AT1G20620,RHC1A,CYP86A8,CYP86A2,CYP86A7	Promote cisplatin-induced apoptosis in gastric cancer	He et al. (2020)
	Limonin	C26H30O8	CYP3A4	Induce apoptosis	Rahman et al. (2015)
*Sparganium stoloniferum* (Buch.-Ham. ex Graebn.) Buch.-Ham. ex Juz. (Typhaceae) (Sanleng)	Gallic acid	C7H6O5	PTGS1,PTGS2,MAOB,PGR,PTPN6,TOP2A,HSP90AB1,PIK3CG,CASP9,CASP3,TP53,FASN,FASLG,MGST1,CYP3A43	Analgesic effects	
	Sparstolonin B	C15H8O5	TLR2,TLR4	A potential therapeutic agent for toll-like receptor–mediated inflammatory disorders	Yepuri et al. (2019); Jin et al., 2018
	Sanleng acid	C18H34O5		Anti-tumor activity	
	β-Elemene	C15H24	PTGS2,GABRA2,RXRANET,CHRM2,GABRA1,GABRA6,PTGS1,CHRM3,CHRM1,ADRA1A,CHRNA7,NCOA2,GABRA5,BCL2,CDKN3,EIF3L,RB1,TP53,TEP1,RUNX1T1,CRK2,CCNB1,RHOA	Analgesic effects; anti-tumor activity; induce apoptosis	Bi et al. (2018); Chen et al. (2021)
*Curcuma phaeocaulis* Valeton (E’zhu)	Curdione	C15H24O2	CYP3A4	Anti-tumor activity	Chen et al. (2021)
	Isoborneol	C10H18O	GABRA6,PGR,CYP2C8,GABRA2,CHRM2,GABRA1,CHRM3,CHRM1,PTGS2,GABRA5,NET,ADRA1A	Anti-inflammatory and analgesic effects	Wang et al. (2017)
	Germacra	C15H24	PTGS2,RXRA,NET,GABRA1,MAOB	Analgesic effects	
	Borneol	C10H18O	CYP2C8,GABRA2,GABRA5,CHRM2,GABRA1,IGHG1,GABRA6,PTGS1,PTGS2,NET	Anti-inflammatory and analgesic effects	Wang et al. (2017)
	Zederone	C15H18O3	NOS2,CHRM3,F2,CHRM1,ADRB2,GABRA1,CHRNA7	Analgesic effects	Faiz et al. (2015)

Furthermore, we had collected 1,289 endometriosis-related genes/targets from GeneCards, GenBank, and OMIM databases. A total of 75 targets of ELC overlapped with endometriosis-related proteins. Information on these targets is provided in Supplementary Figure S5 and Supplementary Table S6. Following cytoHubba analysis, the PPI network revealed that VEGFA, IL6, TP53, PTGS2, AKT1, MMP9, MAPK1, JUN, CASP3, IL10, *etc.*, could be the major relevant targets.

### GO Enrichment and KEGG Pathway Analyses

**FIGURE 2:**
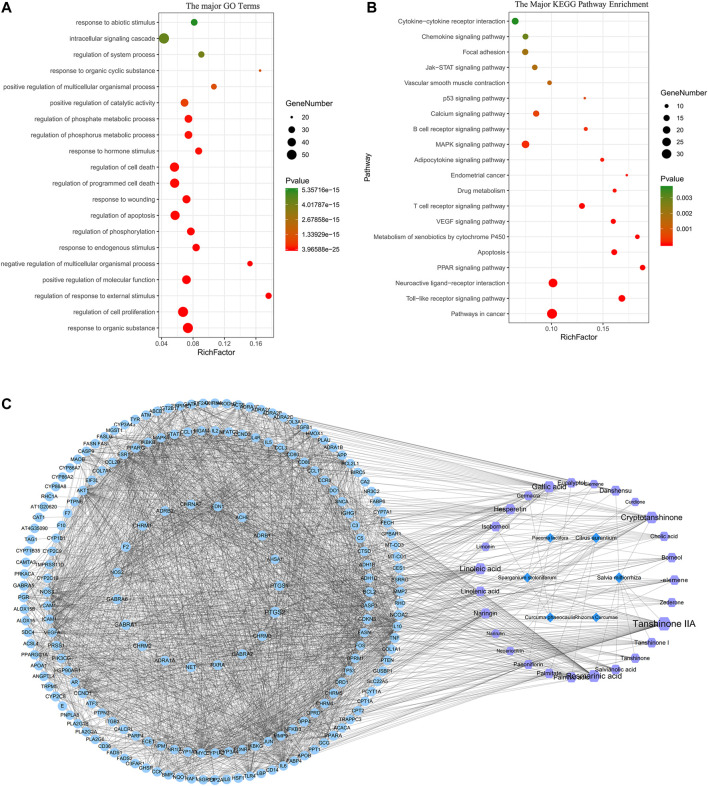
Network pharmacology analysis of ELeng Capsule. **(A)** The major GO terms of BP of potential targets of ELC. **(B)** The 20 major KEGG pathways of potential targets of ELC. **(C)** “Herb–compound–target-signaling pathway” network of ELC. The light blue nodes represent targets (genes); the purple nodes represent compounds; the dark blue nodes represent herbs.

The BP in GO terms is related to cell death, apoptosis, proliferation, *etc.* We also found that some targets are related to the GO terms of smooth muscle hyperplasia and regulation of smooth muscle cell-matrix adhesion. And the main KEGG pathways include signaling molecules and interaction (neuroactive ligand–receptor interaction and cytokine–cytokine receptor interaction), immune system [toll-like receptor (TLR) signaling, B cell receptor signaling, and T cell receptor pathways], and other signal transduction pathways [PPAR signaling pathway, metabolism of xenobiotics by cytochrome P450, vascular endothelial growth factor (VEGF) signaling pathway, and calcium signaling pathway]. Thus, the core compounds of ELC may be involved in regulating inflammation and immunity, reducing adhesion and angiogenesis, and inducing cell apoptosis. The major GO terms and KEGG pathways are shown in Figures 2A,B. The network of major targets and compounds from the database is shown in Figure 2C.

### Potential Mechanism of ELeng Capsule in Endometriosis Rat Model

#### The Effect of ELeng Capsule on Pathology and Ultramicro-Pathology

To further assess the obtained results of network pharmacology analysis, we successfully established a rat model in endometriosis. We mainly examined the effects of ELC in inducing apoptosis and inhibiting angiogenesis and fibrosis.

**FIGURE 3:**
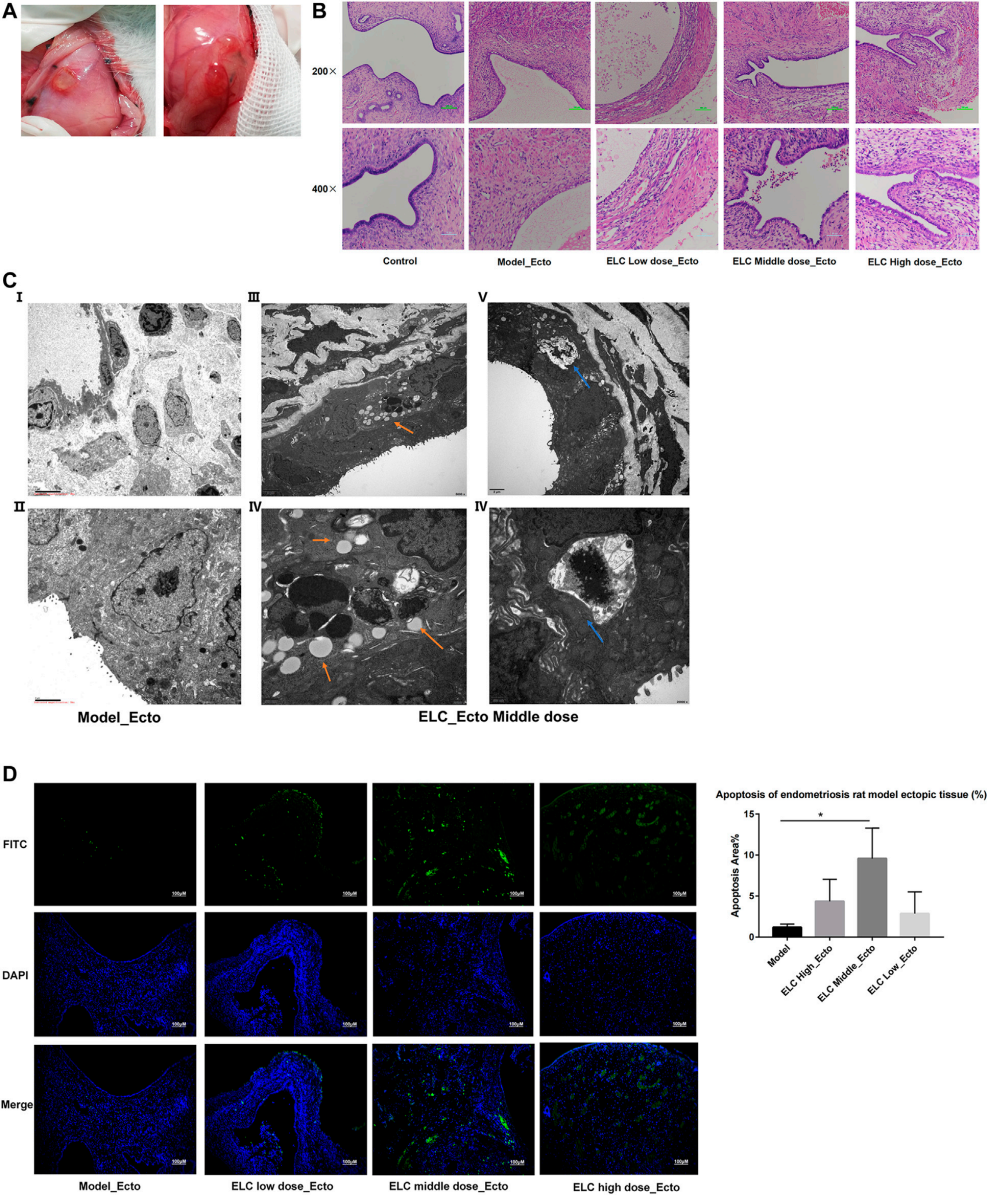
Pathological microstructure and ultrastructure. **(A)** Graphs of ectopic endometrium lesions in endometriosis rat models. **(B)** Microstructure of the ectopic endometrium by HE staining (200× and 400×). **(C)** Ultrastructure of ectopic endometriotic lesions. The blue arrow indicates autophagosome. The orange arrow shows the structure of apoptosis bodies. **(D)** Detection of apoptosis was using the TUNEL assay (100x). Apoptosis in ectopic endometria of different groups was observed by the TUNEL assay. DAPI-stained nuclei appeared in blue. Green-stained tissue appeared in green due to the presence of apoptotic cells. The apoptotic index (%) of ectopic endometrial tissues was significantly higher in middle doze group (*n* = 4).

Figure 3A shows the changes in lesions after modeling. After the treatment, the average value of tissue in ELC groups was lower than that in the model group, while the difference was not statistically significant (*p* > 0.05) (Supplementary Table S7). Compared with that in the model group, the lesion volumes before and after ELC treatment in the ELC middle-dose group changed significantly (*p =* 0.028 < 0.05). These results showed that ELC may reduce the volume of ectopic lesions to a certain extent in endometriosis rat models. The HE staining revealed the formation of local glands in the lesions of the model and ELC group rats (Figure 3B). Compared with the eutopic endometrium in the control group, the ectopic endometrium in the model group had a thinner endometrium, intact glandular epithelial cells, and a loose arrangement.

The ultra-structures have been examined using an electron microscope. In the model group, many microvilli endometrial glandular epithelial cell surfaces and long villi can be seen. In the ectopic tissue in the ELC group, the microvilli were reduced. And the mitochondria were swollen in the cytoplasm, and autophagy and apoptotic bodies were observed (Figure 3C). Figures 3C iii,iv show the apoptotic body, and Figures 3C v,vi show the autophagosome. This result suggested that ELC treatment may be related to the regulation of autophagy and apoptosis.

#### ELeng Capsule Could Promote Apoptosis in Ectopic Endometrial Tissues

The distribution of green fluorescence included glandular epithelial and mesenchymal cells. As shown in Figure 3D, the nuclei of positive staining apoptotic cells emitted green fluorescent signals in the ectopic endometrium tissues. Compared with that in the model group, the apoptotic area in the middle-dose group of ELC increased significantly (*n* = 4, *p* < 0.05). This result suggested that ELC could participate in the process of cell apoptosis.

#### ELC Could Reduce the MVD and the Expression of VEGFA and VEGFC in Ectopic Endometrial Tissues

**FIGURE 4:**
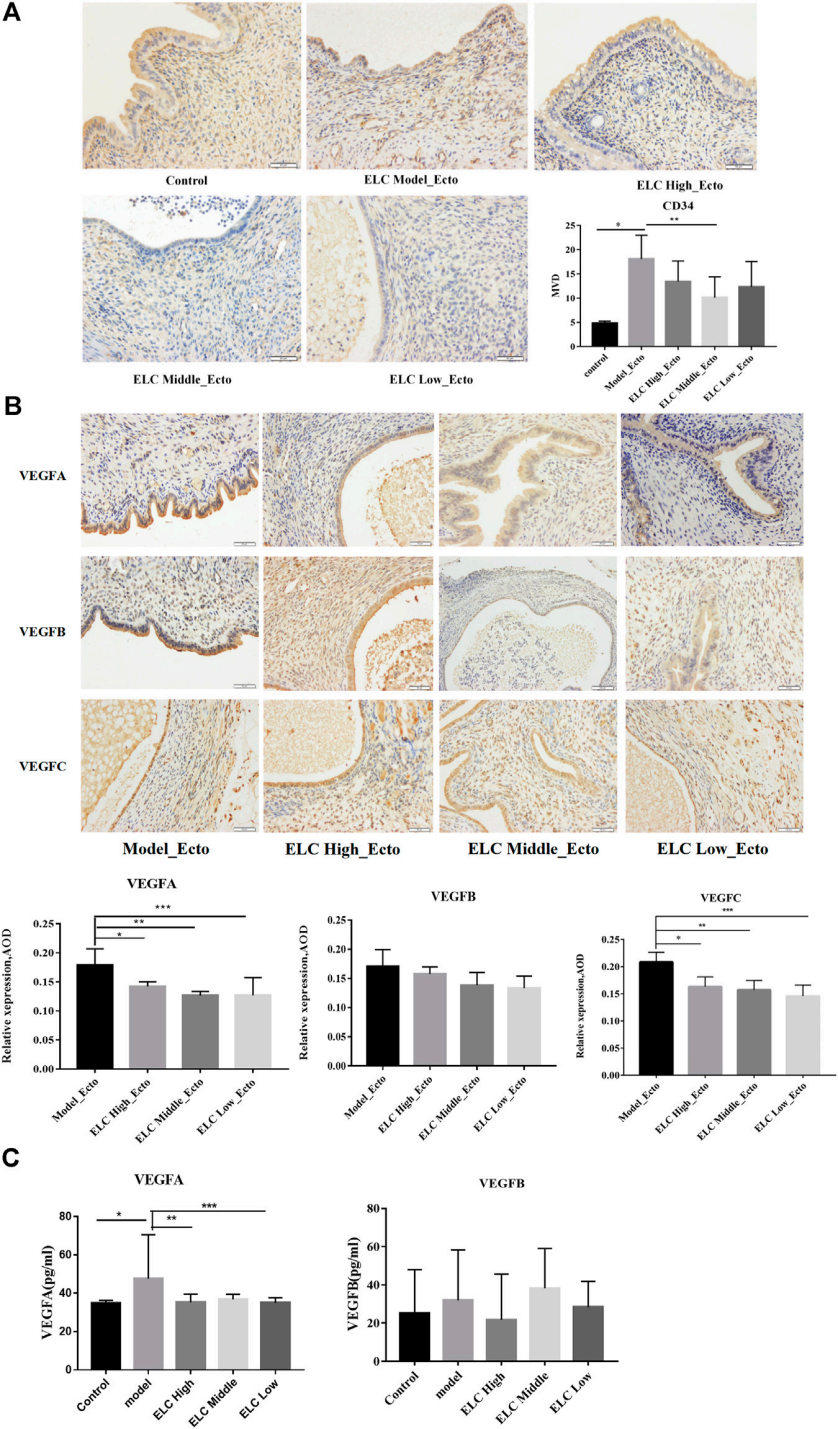
MVD and expression of VEGF in ectopic endometrial tissues. **(A)** Compared with that in the control group eutopic endometrium, the MVD in the ectopic endometrium in the model group increased (*n* = 6, *p = 0.001 < 0.05). Compared with that in the model group, the MVD in the ectopic endometrium in the ELC middle-dose group decreased (**p = 0.018 < 0.05). **(B)** The results suggested that the expression of VEGFA was statistically significant (*p* = 0.014). *p = 0.031 < 0.05; **p = 0.004 < 0.05; ***p = 0.005 < 0.05. There was no significant difference in VEGFB expression among different groups (*p* > 0.05). Compared with that in the model group, the expression of VEGFC was reduced in ELC groups. *p = 0.005; **p = 0.002; ***p = 0.000. **(C)** The expression levels of VEGFA in serum were statistically significant (*F* = 2.742, *p* = 0.044 < 0.05). *p = 0.008; **p = 0.020; ***p = 0.012. There was no significant difference in the expression levels of VEGFB in serum among different groups (*F* = 0.674, *p* = 0.614 > 0.05). Values are represented as mean ± SD, *n* = 4. *p* < 0.05 as determined by one-way ANOVA.

As shown in Figure 4A, a significantly increased MVD was observed in ectopic lesions compared with the corresponding eutopic endometria and normal endometria. The ectopic endometria in the middle-dose group exhibited the highest MVD, and normal endometria exhibited the lowest MVD. The MVD in the ectopic lesion was significantly higher than that in the eutopic endometrium (*n* = 6, *p* = 0.015 < 0.05). The expression of CD34 in high-, middle-, and low-dose groups of ELC was significantly lower than the model group expression.

Figure 4B shows the expression of VEGFA, VEGFB, and VEGFC in the cytoplasm and membrane of glandular epithelial cells and mesenchymal cells in ectopic lesions in the endometriosis rat model. The VEGF gene expressions of mesenchymal cells were weaker than those of glandular epithelial cells. The expression of VEGFA in middle-dose and low-dose groups decreased compared with that in the model group (*p* < 0.05). There was no significant difference between the model group and the ELC high-, middle-, and low-dose groups in the VEGFB expression (*p* < 0.05). Compared with that in the model group, the expression of VEGFC in ectopic lesions significantly decreased in the high-, middle-, and low-dose groups of ELC, and the differences were statistically significant (*p* < 0.05). These results suggested that ELC may inhibit angiogenesis by reducing the expression of VEGFA and VEGFB.

In addition, compared with that in the control group (34.838 ± 1.403 pg/ml, *n* = 8), the VEGFA level in serum increased in the model group (38.866 ± 2.706 pg/ml, *n* = 8) (*p* = 0.008 < 0.05). Compared with that in the model group, the serum VEGFA level in the high-dose group (35.345 ± 4.205 pg/ml, *n* = 8) and low-dose group (35.024 ± 2.662 pg/ml, *n* = 8) of ELC significantly decreased (**p = 0.020, ***p = 0.012). There was no significant difference in the serum of VEGFB expression in different groups of endometriosis model rats (*p* > 0.05). Thus, the regulation effect of ELC may be mainly localized in ectopic lesions (Figure 4C and Supplementary Table S8).

#### ELeng Capsule Could Reduce the Local Fibrosis in Ectopic Lesions

**FIGURE 5:**
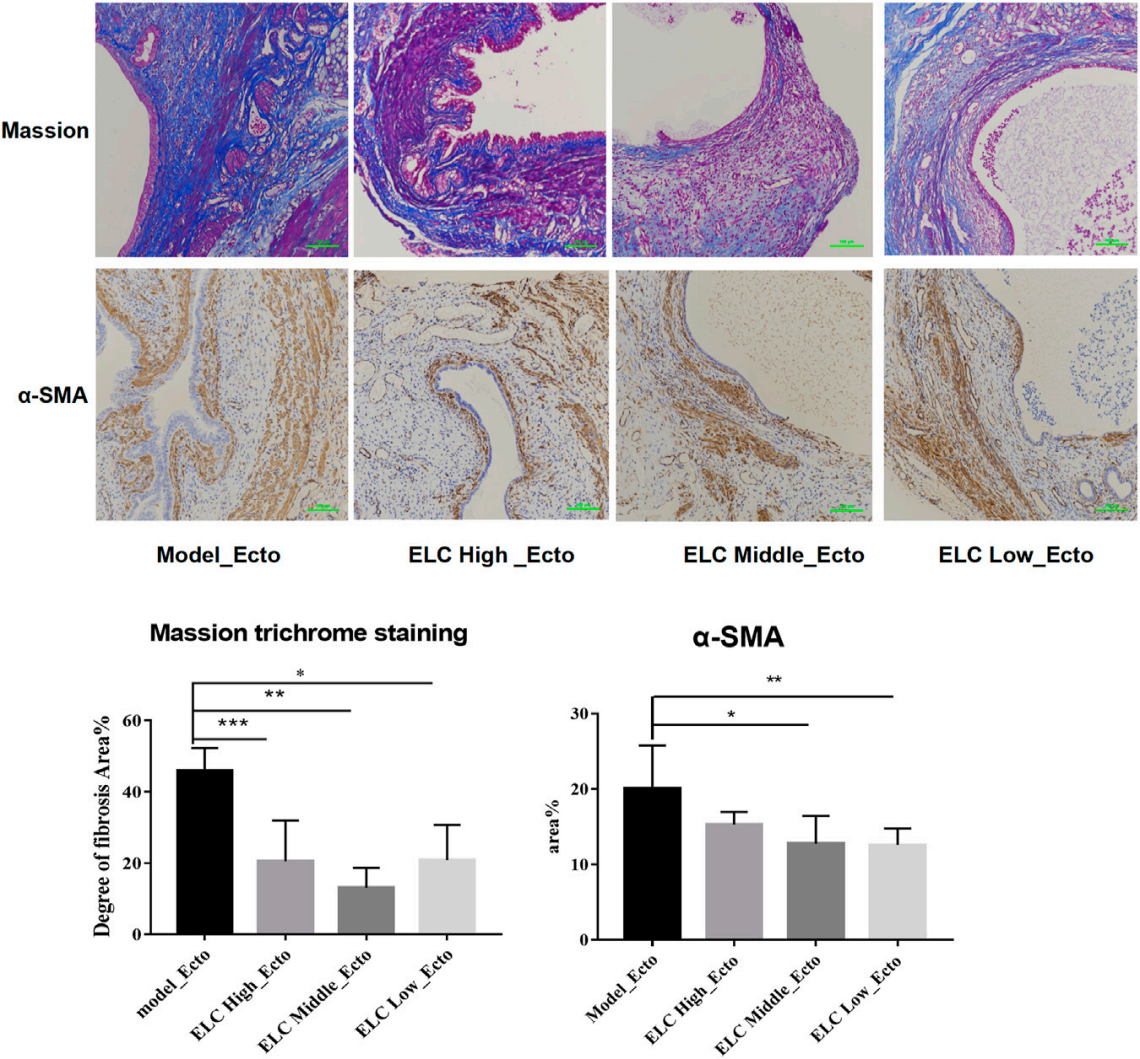
Result of fibrosis in ectopic lesions in endometriosis model rats. Values are represented as mean ± SD, *n* = 4. *p* < 0.05 as determined by one-way ANOVA. The Masson staining showed local fibrosis after modeling (×200). The percentage of fibrosis was positive by Masson staining of ectopic lesions in tissue sections. Compared with the model group, the ELC group has a lower degree of ELC fibrosis. Values are represented as mean ± SD (*n* = 4) (*p < 0.05). ELC could reduce the degree of fibrosis of the lesion. Model group: 45.86 ± 6.42%, ELC High_Ecto: 20.56 ± 11.41%, ELC Middle_Ecto: 13.06 ± 5.68%, and ELC Low_Ecto: 20.87 ± 9.93%. *p = 0.0068; **p = 0.0009; ***p = 0.0074 (*p* < 0.05). Compared with that in the control eutopic endometrium, the fibrosis area (Area%) of the model group and ELC group increased significantly (*p* = 0.0457 < 0.05). Compared with that in the model group, the fibrosis area ratio was reduced in the ELC middle-dose group and low-dose group. *p = 0.040; **p = 0.0346 (*p* < 0.05).

The results of Masson’s trichrome staining showed that the ectopic lesions were fibrotic. Compared with that in the model group, the positive area of fibrosis decreased in the high-, middle-, and low-dose groups of ELC, and the difference was statistically significant (*p* < 0.05). The result showed that ELC could reduce the degree of fibrosis in endometriosis model rats in ectopic lesions. The results of Masson’s trichrome staining are shown in Figure 5. In addition, the expression of α-SMA in ectopic lesions of model rats increased (*p* < 0.05). After ELC treatment, the expression of α-SMA in ELC groups (high-, middle-, and low-dose groups) was all reduced compared with that in the model group (*p* < 0.05). These results suggest that ELC could reduce the fibrosis process of ectopic lesions by inhibiting the expression of α-SMA in ectopic lesions.

### RNA-Sequencing Analysis of Endometriosis Rat Model Characteristics and the Treatment With ELeng Capsules

#### The Differentially Expressed Gene Screening Analysis

We further analyzed the potential mechanism of ELC by RNA transcriptome. According to the results of the principal components analysis, there is no difference in Con_Euto, Model_Euto, and ELC_Euto groups. These suggested that ELC may not affect the eutopic endometrium in endometriosis rat models. There were a total of 1,461 DEGs in Con_Euto vs. Model_Ecto groups, 557 DEGs in Model_Ecto vs. ELC_Ecto groups, and 1,097 DEGs in Con_Euto vs. ELC_Ecto groups (FC-1.5). Supplementary Figure S6 shows the PCA of five groups (A), Venn analysis of more than five groups (B), upregulated and downregulated DEGs (C), and heatmap illustration (D).

In this study, there were 1,048 upregulated DEGs and 413 downregulated DEGs between Model_Ecto and Con_Euto groups. These DEGs mainly participate in the processes such as inflammation, cytoskeleton, EMT, and angiogenesis. In the ELC_Ecto *vs.* Model_Ecto group analysis, a total of 66 and 491 upregulated and downregulated DEGs, respectively, were identified, reflecting the differential expression of related genes after treatment with ELC.

#### GO and KEGG Enrichment Analyses of Model_Ecto vs. Con_Euto

**FIGURE 6:**
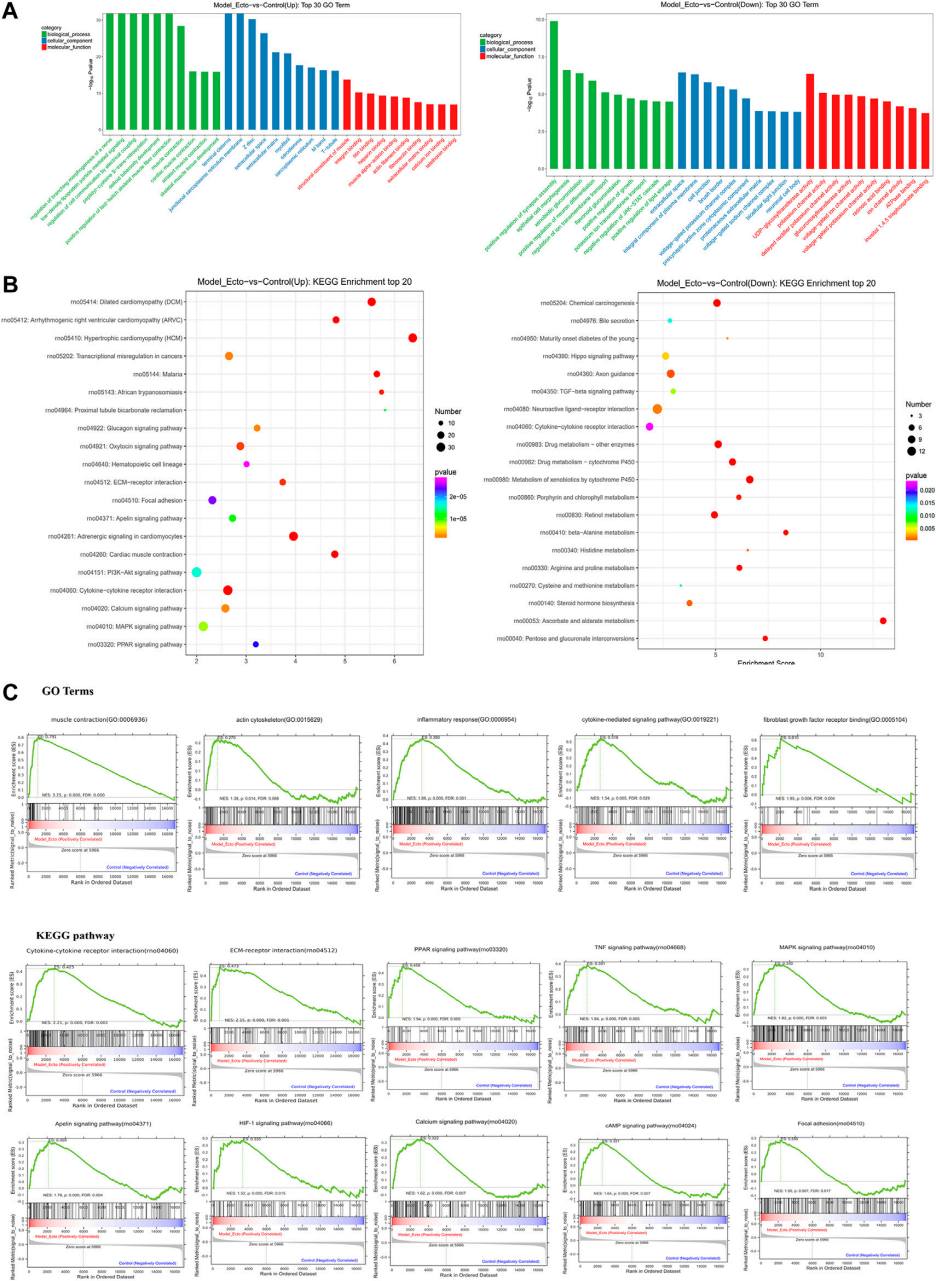
RNA-sequencing reveals the characteristic of endometriosis rat models induced by autotransplantation. Transcriptome characteristics of endometriosis model rats were found through comparing the ectopic endometrium in model rats and the eutopic endometrium in control group rats. **(A)** Results of GO enrichment analysis for upregulated DEGs and downregulated DEGs reversed by ELC ectopic endometrium groups and model ectopic endometrium groups. **(B)** KEGG analysis of upregulated and downregulated genes. KEGG pathway analysis of upregulated and downregulated genes in the ectopic endometrium in rat models *vs.* control eutopic endometrium. The gene ratio refers to the ratio of the number of target genes associated with a KEGG pathway to the total number of genes in the pathway. **(C)** GSEA in endometriosis rat models showed enrichment of GO analysis and KEGG pathway. The normalized enrichment score (NES), *p*-value, and false discovery rate (FDR) are indicated for each gene set. GSEA revealed a significant enrichment of gene signatures associated with endometriosis (*p* < 0.05).

We analyzed the characteristics of the rat endometriosis model based on our RNA-sequence data based on the DEGs of Model_Ecto *vs.* Con_Euto. As shown in GO terms, the upregulated genes were most significantly enriched in the CC of extracellular region, the BP of muscle contraction, and the MF of actin filament binding, fibronectin binding, calcium ion binding, *etc*. The actin-associated GO terms may relate to the development of ectopic lesions of endometriosis (Figure 6A). The major upregulated KEGG analysis pathways were extracellular matrix–receptor (ECM–receptor) interaction, p53 signaling pathway, endocrine resistance, interleukin-17 (IL-17) signaling pathway, chemokine signaling pathway, cytokine–cytokine receptor interaction, *etc.* (Figure 6B).

Furthermore, utilizing data from the GeneCards dataset, we found that there were 113 upregulated and 28 downregulated DEGs in Model_Ecto *vs.* Con_Euto overlapped with endometriosis genes. The upregulated genes associated with endometriosis in humans are related to cytokine–cytokine receptor interaction, phosphatidylinositol 3 kinase–Akt (PI3K–Akt) signaling pathway, pathway in cancer, MAPK signaling pathway, ECM–receptor interaction, Ras signaling pathway, toll-like receptor (TLR) signaling pathway, IL-17 signaling pathway, p53 signaling pathway, forkhead box protein O signaling pathway, focal adhesion, etc. Based on the above analyses, the rat model of endometriosis may be suitable for investigating the transcriptome level.

In addition, based on GSEA, neuroactive ligand–receptor interaction, cell adhesion molecules, and regulation of actin cytoskeleton are closely related to the occurrence and development of endometriosis. The development of endometriosis is related to the GO terms of “skeletal muscle fiber,” “endodermal cell differentiation,” “regulation of signaling receptor activity,” “positive regulation of myoblast differentiation,” “response to cytokine,” “chemokine-mediated signaling pathway,” “positive regulation of smooth muscle cell migration,” etc. The KEGG pathways of GSEA showed that the peroxisome proliferator–activated receptor signaling, tumor necrosis factor signaling, MAPK signaling, apelin signaling, hypoxia-inducible factor-1 signaling, PI3K–Akt signaling pathway, and focal adhesion are related to endometriosis (Figure 6C) (*p <* 0.01, FDR <0.25).

#### GO and KEGG Enrichment Analyses of ELC_Ecto vs. Model_Ecto

**FIGURE 7:**
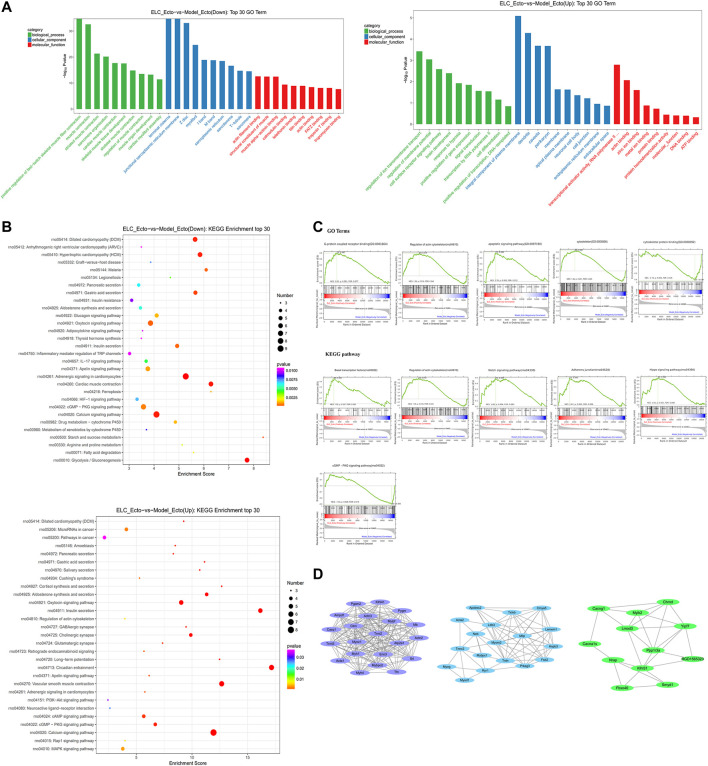
RNA-sequencing reveals the transcriptome profile of gene expression changes in the treatment with ELC. The related genes and pathways regulated by ELC were found through comparing the DEGs between the Model_Ecto group and the ELC_Ecto group. **(A)** Results of gene ontology enrichment analysis for upregulated DEGs and downregulated DEGs reversed by ELC ectopic endometrium groups and model ectopic endometrium groups. **(B)** Results of KEGG enrichment analysis between ELC_Ecto groups and Model_Ecto groups. **(C)** The results of GSEA revealed a significant enrichment of gene signatures associated with ELC treatment (*p* < 0.05). **(D)** MCODE network of cluster analysis of the potential targets network in ELC treatment.

We mainly focused on the DEGs in ectopic lesions of endometriosis rats after the intervention of ELC based on DEGs of ELC_Ecto *vs.* Model_Ecto. The BP, CC, and MF GO terms suggested muscle- and troponin-associated regulation, which could be related to ELC treatment. The major enriched GO BPs were positive regulation of fast-twitch skeletal muscle fiber contraction, muscle contraction, striated muscle contraction, etc. The major enriched GO CCs were terminal cisterna, junctional sarcoplasmic reticulum membrane, Z disc, etc. The major enriched GO MFs were actin filament binding, structural constituent of muscle, actin binding, etc. (Figure 7A). These results revealed that the treatment with ELC could be related to the regulation of troponin and cytoskeleton.

We further analyzed the KEGG pathways of ELC_Ecto vs. Model_Ecto. The downregulated DEGs were mainly enriched in the following pathways: calcium signaling, apelin signaling, cyclic guanosine monophosphate–protein kinase G (cGMP–PKG) signaling, 5′ adenosine monophosphate–activated protein kinase signaling, hypoxia-inducible factor-1 (HIF-1) signaling, MAPK signaling, PI3K–Akt signaling pathway, focal adhesion, *etc.* (Figure 7B).

Based on GSEA, we also found other signaling pathways, including the Notch signaling pathway, adherens junction, Hippo signaling pathway, and regulation of actin cytoskeleton, which were related to the treatment of endometriosis with ELC (FDR <0.25) (Figure 7C). ELC may inhibit fibrosis and EMT by regulating the aforementioned pathways. The core genes established in the network may be related to the regulation by ELC treatment. Following MCODE analysis in Cytoscape, we selected three major modules for module network visualization (Figure 7D). The core nodes continued to be associated with genes related to actin, cytoskeleton, and fibrosis.

#### STEM Analysis of Differential Expression Patterns

**FIGURE 8:**
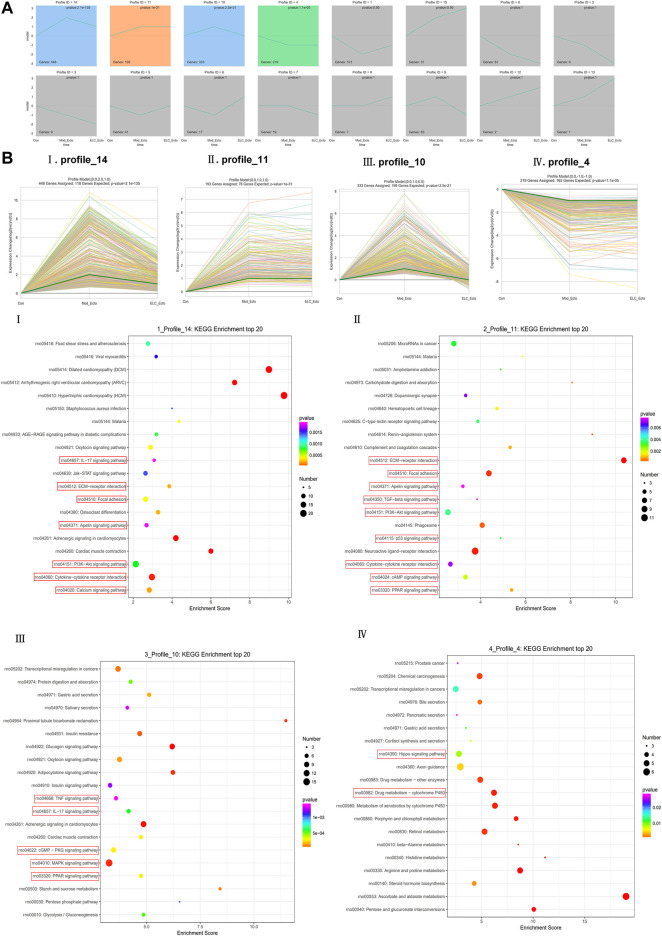
STEM analysis of ELC treatment in endometriosis rats. **(A)** Trend chart of overall STEM analysis. **(B)** Four statistically significant trends. The results of gene cluster analysis were statistically significant in profiles 14, 11, 10, and 4 (*p* < 0.05). The profile_11 panel **(Bii)** and profile_4 panel **(Biv)** could be the regulatory genes of endometriosis model development (*p* < 0.05). The series test of the profile_14 panel **(Bi)** and profile_10 panel **(Biii)** showed that the significant clusters were considered potential profiles that could be affected by ELC treatment (*p* < 0.05). **(i)** *p* = 2.1E-135, **(ii)** *P* = 1E-31, **(iii)** *p* = 2.50E-21, and **(iv)** *p* = 0.000011.

The results of the gene cluster analysis were statistically significant in profile_14, profile_11, profile_10, and profile_4 (*p <* 0.05) (Figure 8A). After the development of the endometriosis model, actin-associated DEGs in the Model_Ecto and ELC_Ecto groups were upregulated; these may be the regulatory genes for the development of the endometriosis model (profile_11 and profile_4). The series test of profile_14 and profile_10 showed that the significant clusters were considered potential profiles that could be affected by treatment with ELC (*p <* 0.05). Several actin-related and microfilament proteins were upregulated in the model group and downregulated after treatment, suggesting that the overall regulation mechanism of ELC treatment in the ectopic endometrium is related to the regulation of actin cytoskeleton (Figure 8B).

#### Protein–Protein Interaction Network

**FIGURE 9:**
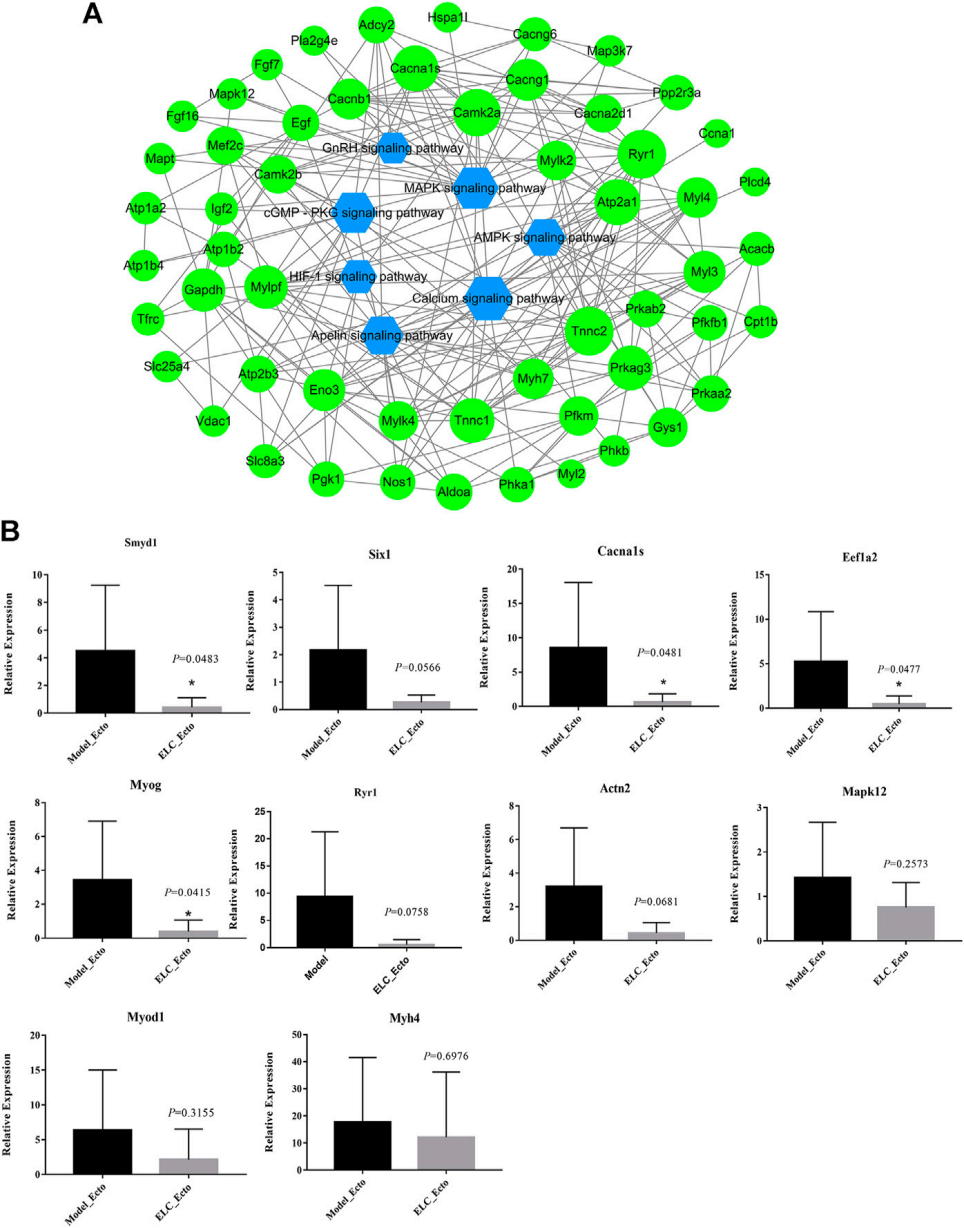
DEG effect of ELeng Capsules in the endometriosis model rats. **(A)** Network of major KEGG pathways and targets of Model_Ecto vs. ELC_Ecto downregulated DEGs. **(B)** Expression of genes in ectopic endometrium tissues in endometriosis rat models detected by qRT-PCR and shown by the expression fold changes. ACTB was used as the internal control. Data are shown as mean ± SD, *p < 0.05.

We explored the relationship between the endometriosis-related genes and downregulated genes after treatment with ELC. We constructed network relationships between the core genes of the two groups of DEGs and analyzed the possible network relationships through relevant pathways. We selected the calcium signaling pathway, cGMP–PKG signaling pathway, apelin signaling pathway, HIF-1 signaling pathway, AMPK signaling pathway, GnRH signaling pathway, and associated DEGs to construct the network (Figure 9A). The hub downregulated genes closely related to treatment with ELC were as follows: ACTN3, ACTN2, MYOM2, myoglobin, RYR1, MYOG, MYH7, MYOD1, sarcalumenin, myosin light chain kinase 2 (MYLK2), SMYD1, MAP3K7, MAPK12, MYH4, CACNA1S, EEF1A2, and CACNG1.

### Identifying the Potential Genes in ELC Treatment

The expression ratios of these DEGs are determined by qPCR (Figure 9B and Supplementary Table S9). The genes, which are related to tumors, cytoskeleton, and cell potential, have numerous biological functions and may be involved in the development of endometriosis. EEF1A2 encodes an isoform of the alpha subunit of the elongation factor-1 complex and may be critical in the development of ovarian cancer (Worley et al., 2015). Targeting EEF1A2 and plitidepsin to release protein kinase R may trigger the extrinsic pathway of MAPK and nuclear factor-κB–dependent activation, leading to tumor cell death (Losada et al., 2018). RYR1 is the core factor of the calcium signaling pathway. The ryanodine receptor calcium release channel is central to the cytoplasmic calcium signaling pathway (Dulhunty et al., 2018).

## Discussion

Endometriosis is a common and difficult gynecological disease. Even now, its exact mechanisms are still not clearly understood, and treatment strategies still need to be further improved. A growing body of evidence showed that the mechanism of Chinese medicine in the treatment of endometriosis could be related to inhibiting inflammation, enhancing the immune response, regulating angiogenesis-related pathways, and inducing apoptosis (Weisheng et al., 2019). Overall, ELC has the benefits of activating blood circulation, removing blood stasis, and relieving pain. In this study, we investigated the regulated genes of ELC by network pharmacology and RNA-sequencing and found the characteristics of a rat model of endometriosis as well.

### The Characteristics of Endometriosis Rat Model

In the present study, we compared the expression of genes in endometriotic lesions in a rat model and the eutopic endometria of normal rats by RNA-sequence. We found that upregulated DEGs between Model_Ecto and Con_Euto were significantly enriched in several pathways, including focal adhesion, ECM–receptor interaction, calcium signaling pathway, and cytokine–cytokine receptor interaction. The results of RNA-seq indicate the EMT and fibrosis in ectopic endometrium lesions in endometriosis. Another study of the rat endometriosis model suggested that osteopontin, Lyn, Vav1, Runx1, and l-selectin play important roles in the pathogenesis of endometriosis based on gene expression profiling (Konno et al., 2007).

EMT and fibroblast-to-myofibroblast transdifferentiation as well as increases in cellular contractility, collagen production, and smooth muscle metaplasia lead to fibrosis (Zhang et al., 2016; Liu et al., 2018). These pathological changes may be triggered by infection, mechanical damage, and inflammation and induce EMT in the mesothelium (Albertsen and Ward, 2017). Endometriotic tissue is often induced in rodents via transplantation through surgery or intraperitoneal injection of uterine tissue fragments. The time of collection in rat models is 8 weeks after modeling in our study and the lesion has begun to undergo fibrosis. Thus, the model could reflect the fibrosis and EMT characteristics of endometriosis.

Furthermore, tissue remodeling genes in cytoskeleton, smooth muscle contraction, cellular adhesion, tight junctions, and O-glycan biosynthesis were the most significant to lesions (Sohler et al., 2013). The roles of actin and cytoskeleton in the development of endometriosis, as well as the relationship with cell adhesion, invasion, and fibrosis (Zhan et al., 2016), also attracted our attention.

In summary, the rat models of endometriosis could represent the characteristics of endometriosis to a certain extent (Gu et al., 2020) and could contribute to the molecular pathology of peritoneal endometriosis. Although animal models cannot completely recapitulate the human disease process, they could help understand the complex and interactive roles of the endometrial phenotype, the peritoneal microenvironment, and pathogenic genes, which collectively determine an individual’s risk of developing endometriosis (Bruner-Tran et al., 2018).

### The Potential Mechanism of ELC Treatment

In this study, we had identified 40 compounds in ELC, established the compounds and targets network, and performed further analysis of the potential mechanism involved in treatment with ELC by network pharmacology and RNA-sequence. Interestingly, we found that compounds in ELC could relieve endometriosis-associated pain and regulate the neuroactive ligand–receptor interaction, metabolism of xenobiotics by cytochrome P450, and TLR signaling, VEGF signaling, and calcium signaling pathways. Furthermore, some targets belonged to more than one compound, which suggested that these uniform targets might be the foundation of synergistic therapeutic effect.

The reported efficacy of compounds is related to the mechanism of ELC in endometriosis treatment. Previous phytochemical investigations indicated that the main constituent of *C. phaeocaulis* and *S. stoloniferum* could present anti-tumor and anti-inflammatory activity. Sparstolonin B could serve as a potential therapeutic agent for the treatment of TLR-mediated inflammatory disorders (Yepuri et al., 2019) and also alleviate neuropathic pain by selectively suppressing TLR2 and TLR4 (Jin et al., 2018). β-Elemene, a terpenoid from *C. phaeocaulis*, possesses broad-spectrum anti-tumor activity and is effective against several types of tumors (Bi et al., 2018). Borneol and isoborneol are the monoterpenoid compounds with effective anti-inflammatory and analgesic effects (Wang et al., 2017). Zederone as an analgesic principle could be used to relieve pain in rheumatic disorders in mice (Faiz et al., 2015). The above compounds are suggested to be the effective compounds for the analgesic effect in ELC. Furthermore, *C. phaeocaulis*– and *S. stoloniferum*–medicated serum might suppress TGF-β1–induced EMT in triple-negative breast cancer by decreasing the phosphorylated Smad3 pathway *in vitro* (Yin et al., 2018). *C. phaeocaulis* and its terpenoids (β-elemene, germacrone, curdione) could be the potential anti-cancer drugs (Chen et al., 2021).

*S. miltiorrhiza* and *P. lactiflora* Pall*.* are the herbal medicine that has long been used for the treatment of blood stasis and dysmenorrhea. S. *miltiorrhiza* has the effects of promoting blood circulation, eliminating blood stasis, and relieving pain. The active compounds of *S. miltiorrhiza* include tanshinone I, tanshinone IIA, salvianolic acid, and dihydrotanshinone (MEIm et al., 2019). Salvianolic acid A has several pharmacological actions such as anti-thrombosis and anti-fibrosis (Xu et al., 2018). Tanshinone IIA could reduce the VEGF/VEGFR2 pathway and CD146 *in vitro* and *in vivo* and regulate angiogenic function in human umbilical vein endothelial cells (Zhang et al., 2017). Tanshinone IIA could also improve the paw withdrawal threshold to reduce the mechanical hyperalgesia and regulate the DRG renin angiotensin system (RAS) by reducing the protein expression of AGT, REN, ACE, ANGII, and AT2 in DRG neurons (Chen and Gong, 2020). Tanshinone IIA could also inhibit ectopic endometrial stromal cell (EESC) proliferation and migration through the extracellular matrix (ECM)–receptor interaction pathway and estrogen signaling pathway based on iTRAQ analysis (Luo et al., 2021). Rosmarinic acid is a potential natural compound with anti-cancer properties, as demonstrated in various human cancer cell lines (Yesil-Celiktas et al., 2010). Cryptotanshinone could enhance anti-tumor activity by targeting STAT3-related receptors and targeting NF-κB–related pathways (Wu et al., 2020). It also could inhibit the proliferation of primary HESCs and T-HESCs and induce cell cycle arrest of the latter in the G2/M phase *in vitro* (Ferella et al., 2018). *P. lactiflora* Pall. has hematopoietic functions, anti-inflammatory activity, and immunological properties. In *P. lactiflora*, paeoniflorin exerts anti-inflammatory effect through multiple targets (Zhou et al., 2020) and inhibits the plantar incision–induced microglia TLR4/MMP-9/2/IL-1β signaling pathway and suppresses postoperative pain (Fan et al., 2018). Paeoniflorin, as the major compound in Guizhi Fuling prescription, might play a critical role in the anti-endometriosis effect based on the gray correlation analysis strategy (Chen et al., 2020). Furthermore, paeoniflorigenone could induce apoptosis and suppress proliferation (Huang et al., 2017).

Furthermore, the use of *C. aurantium* is mainly focused on improvement of qi stagnation and remission of pain. And *C. aurantium* may possess anti-tumor activity as well. Naringenin could induce apoptosis and endoplasmic reticulum stress through regulation of the MAPK and Akt signal transduction pathways in End1/E6E7 and VK2/E6E7 cells (Park et al., 2017). Limonin could induce apoptosis, thereby affecting the growth of SNU449 and HCT-15 tumor cells (Rahman et al., 2015). Hesperetin could promote cisplatin-induced apoptosis in gastric cancer through upregulating the expression of tensin homolog (PTEN) (He et al., 2020). Naringin and its aglycone naringenin have shown anti-carcinogenic activities through cell signal transduction pathways in cancer (JAK–STAT pathway, PI3-kinase/Akt/mTOR pathway, Notch pathway, NF-κB and cox-2 pathway, Wnt pathway, MAPK-ERK pathway, TGF-β pathway) (Memariani et al., 2020). In summary, ELC has the characteristic of multi-target regulation, which may regulate angiogenesis and induce apoptosis based on network pharmacology. Our experimental validation also provides evidence of these effects.

These compounds of ELC may exert new synergistic regulatory effects. In order to further explore the regulatory mechanism of ELC in the transcription level, we further conducted RNA-sequence analysis. The results suggested that the DEGs are related to the cytoskeleton, EMT, fibrosis, muscle fibrosis, and MAPK signaling pathway after ELC treatment, which expanded our understanding of the regulatory effect of ELC. Interestingly, we found that the transcriptome analysis and network pharmacology only partially overlap. And this discrepancy may be related to the comprehensive regulation of a variety of compounds, drug responses of experimental animals, differences in regulation of transcription and translation levels, *etc*. The comprehensive regulation mechanism of herbal medicine still needs to be further studied.

Regulation of cytoskeleton and EMT process is also one of the important approaches for endometriosis treatment. These pathological processes are more closely related to abdominal endometriosis and deep infiltration of endometriosis patients (Ping et al., 2016). In addition, through the GSEA of the ELC_Ecto group and the Model_Ecto group, the results of the KEGG enrichment analysis revealed a relationship with the Notch signaling pathway and the Hippo signaling pathway. The hyperactivation of the ADAM17/Notch signaling pathway could result in an increase in fibrosis, which is associated with deep infiltrating endometriosis (DIE) (Gonzalez-Foruria et al., 2017).

**FIGURE 10:**
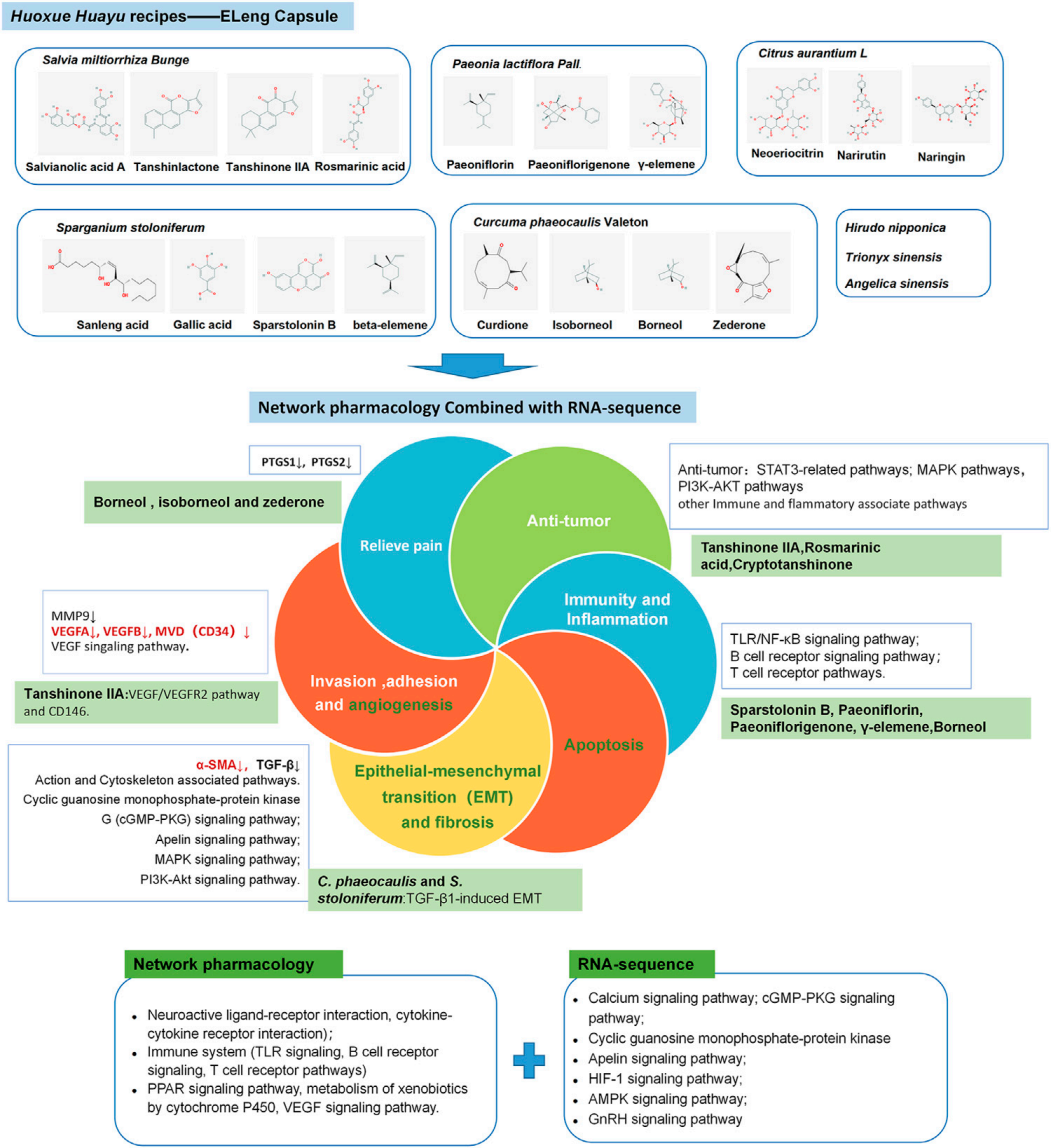
Potential multiple target mechanisms of ELeng Capsule.

In other enriched KEGG pathways of ELC regulation, apelin, as a ligand of the APJ receptor, has functions in angiogenesis and cell proliferation and is a vasoactive and regulatory peptide (Luo et al., 2018). And apelin expression in the eutopic and ectopic endometria changes periodically (Ozkan et al., 2013). The DEGs in the apelin signaling pathway are related to muscle contraction, calmodulin binding, and the myosin complex. Moreover, the kinase-associated pathways are associated with endometriosis. A genome-wide association study analysis revealed that multiple pathways, new variants in MAP3K4, and several pathways linked to MAPK are associated with endometriosis (Uimari et al., 2017). The serine/threonine kinase Akt and extracellular regulatory kinase signaling pathways can synergistically support deep endometriosis by enhancing the proliferation and survival of endometrial stromal cells (ESCs) in the *in vitro* fibrotic microenvironment (Matsuzaki and Darcha, 2015). The above-mentioned pathways, as related pathways for endometriosis, may participate in the regulation process of ELC. The potential mechanism of ELC is shown in Figure 10.

Based on this research, we also have a new discovery about the mechanism of action of Chinese medicine for removing blood stasis. At present, current research on the role of TCM in promoting blood circulation and removing phlegm is focused on apoptosis, inflammatory immunity, and angiogenesis in endometriosis. These results suggest that endometriosis is associated with EMT and that there are differences in differentially expressed proteins among various syndromes in TCM (Wen et al., 2018). Several natural compounds suggested the treatment of cancer, inflammatory, and fibrosing diseases through the regulation of the EMT process (Avila-Carrasco et al., 2019). The mechanism for the regulation of cytoskeleton and EMT through TCM is lacking. And the mechanism of ELC on EMT and fibrosis needs further investigation in terms of compound, single herb, and prescription optimization.

### Limitation

There are several limitations in this study. Firstly, in the HPLC/GC-MS analysis of TCM, only the small molecule compounds derived from plants in ELC were analyzed. The three source animals were not analyzed. Secondly, we obtained our results using rat endometriosis models. Although we have shown that rats/mice are a good animal model for studying endometriosis, they cannot reflect the natural course of the human disease. Further research on cells is warranted to clarify the mechanism involved in the intervention with ELC and study the relationship between the regulation of the cytoskeleton and troponin and the presence of endometriosis.

## Conclusion

In this study, we have explained the treatment mechanism of ELC using transcriptome analysis and network pharmacology. We hypothesized that ELC may regulate inflammation, immunity, cell adhesion, and cytoskeleton-related genes, influence the process of EMT, and consequently affect the development of lesions. Combined techniques may also offer an efficient method of drug discovery from herbal medicine.

## Data Availability

The raw data supporting the conclusions of this article will be made available by the authors, without undue reservation, to any qualified researcher.

## Abbreviations

BP, biological process; CC, cellular component; DEGs, differentially expressed genes; DL, drug-likeness; GO, gene ontology; KEGG, Kyoto Encyclopedia of Genes and Genomes; MCODE, Molecular Complex Detection; MF, molecular function; OB, oral bioavailability; PCA, principal component analysis; PPI, protein–protein interaction; QC, quality control; RT-PCR, real-time polymerase chain reaction; TCMSP, traditional Chinese medicine systems pharmacology; UPLC-Q-TOF/MS, ultraperformance liquid chromatography with quadrupole time-of-flight mass spectrometry.
