# Supplementary figures and images for: Exploration of the Modulatory Property Mechanism of ELeng Capsule in the Treatment of Endometriosis Using Transcriptomics Combined With Systems Network Pharmacology
Source: Front Pharmacol. 2021 Jun 18;12:674874. doi: 10.3389/fphar.2021.674874 (PMC8249582; doi:10.3389/fphar.2021.674874)

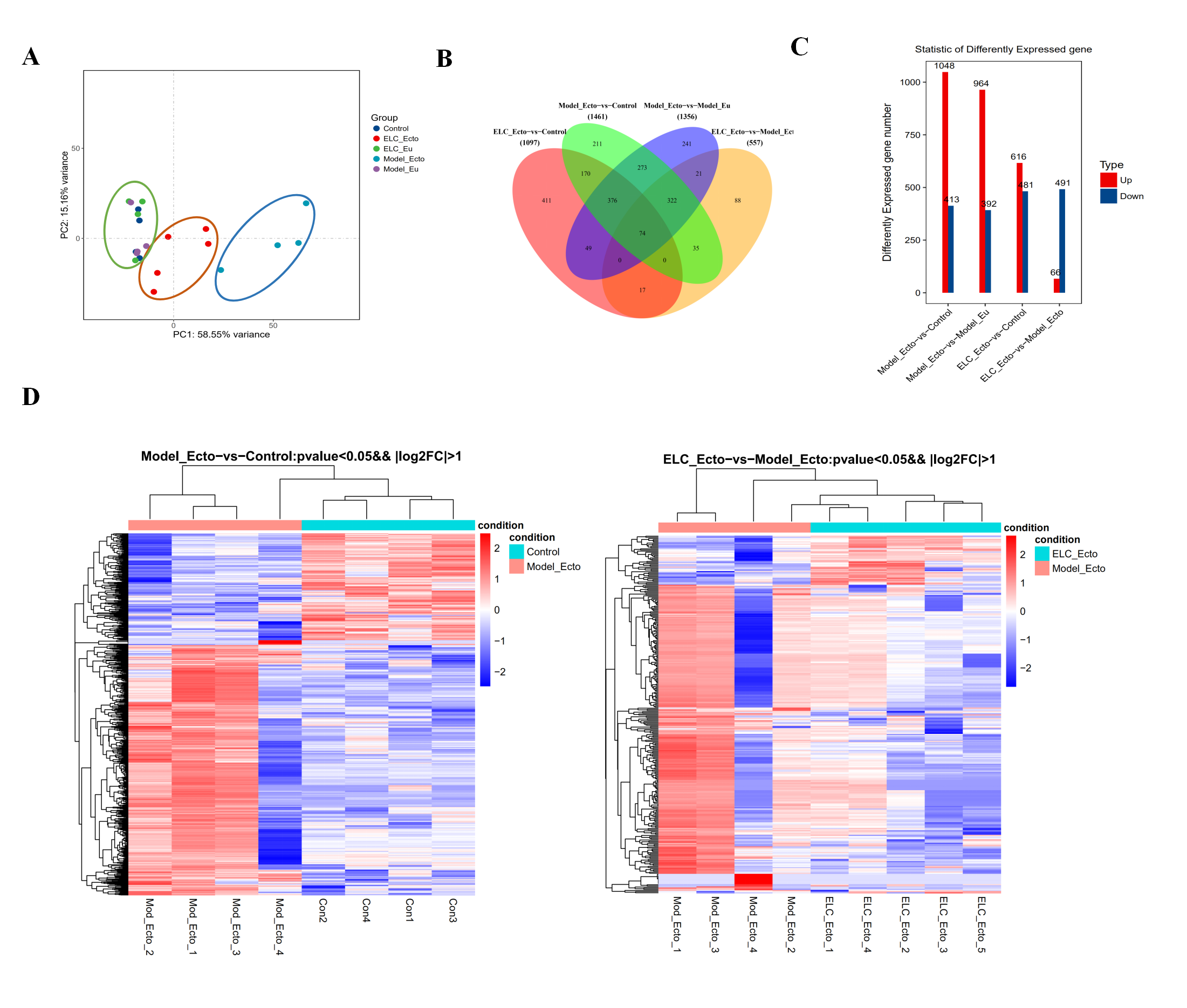

Supplement: Supplementary file 1 [file Image6.TIF]

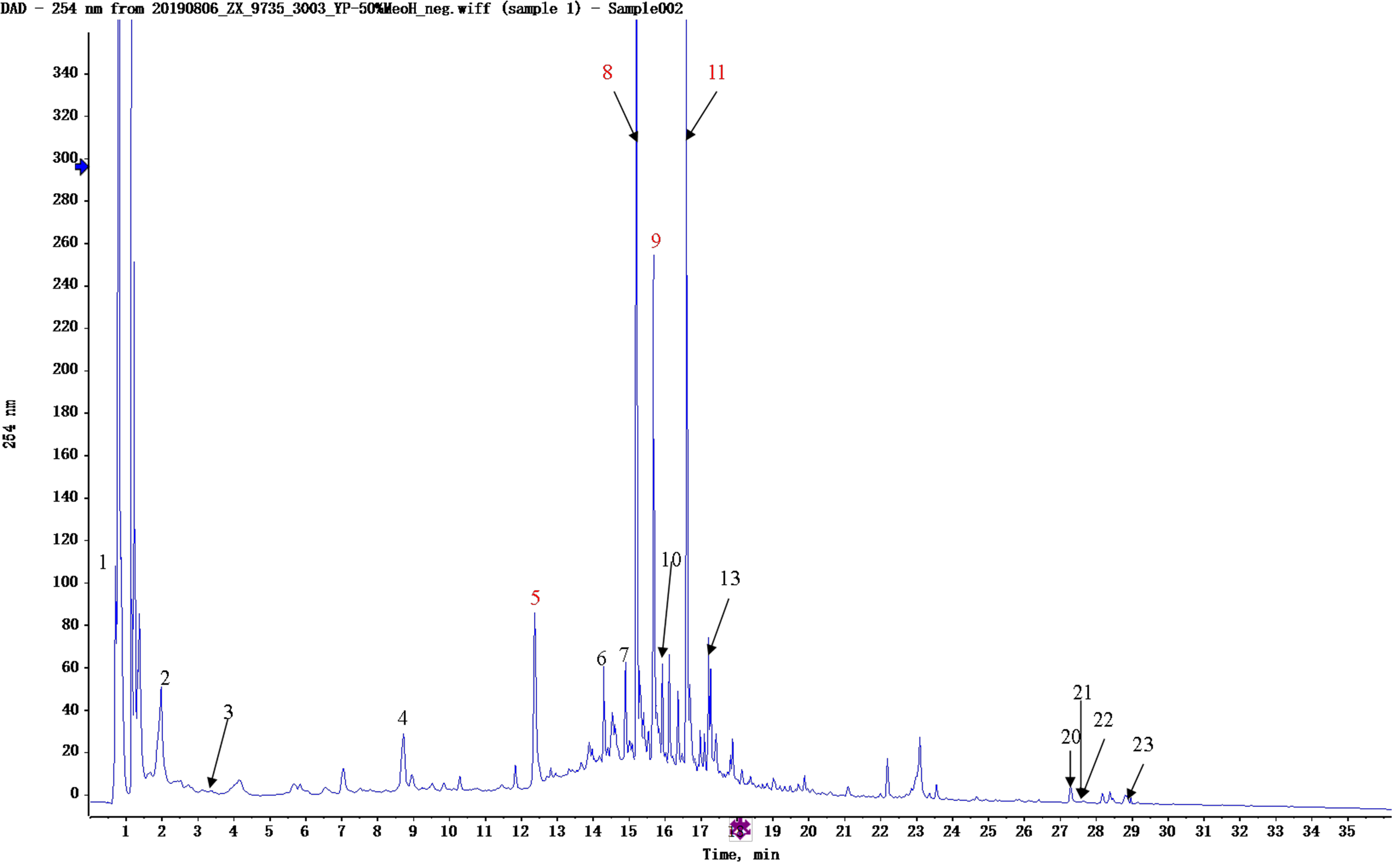

Supplement: Supplementary file 4 [file Image3.TIF]

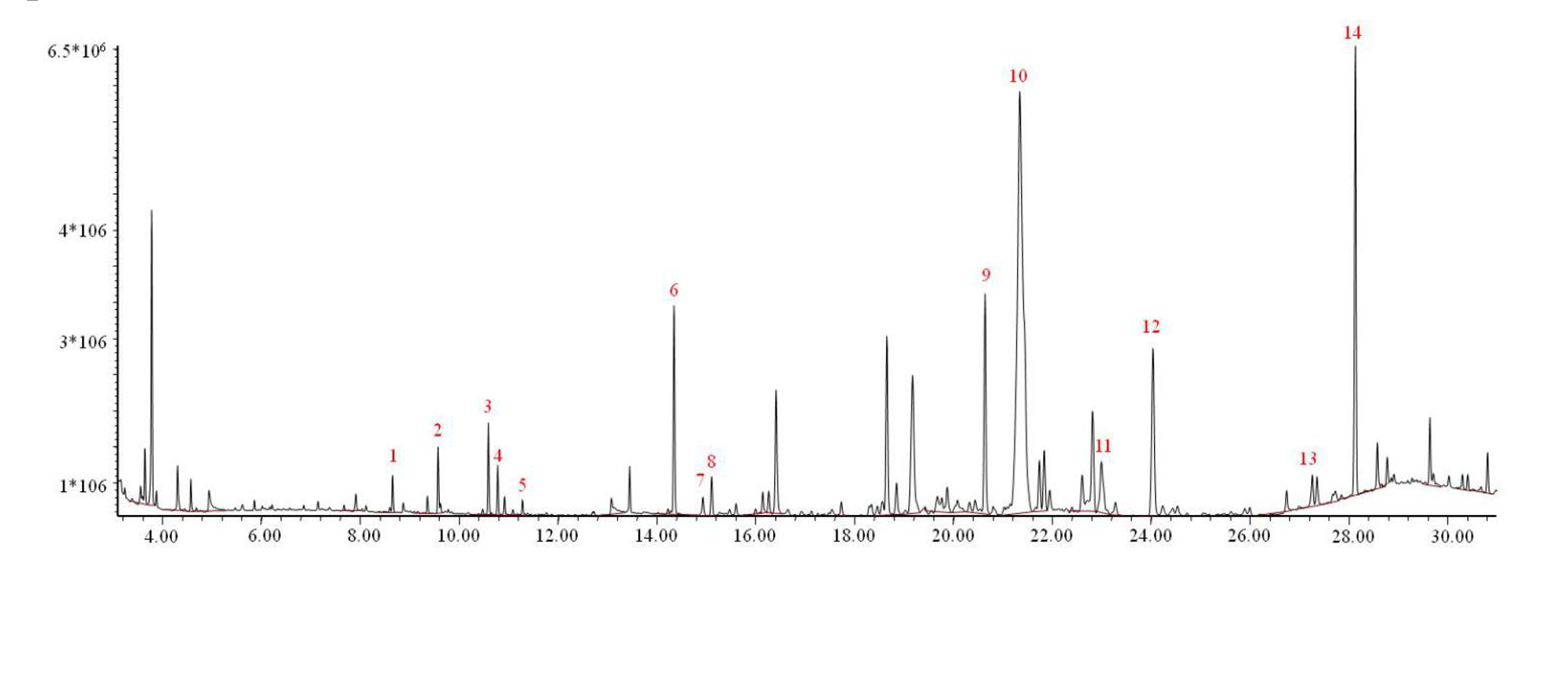

Supplement: Supplementary file 5 [file Image4.TIF]

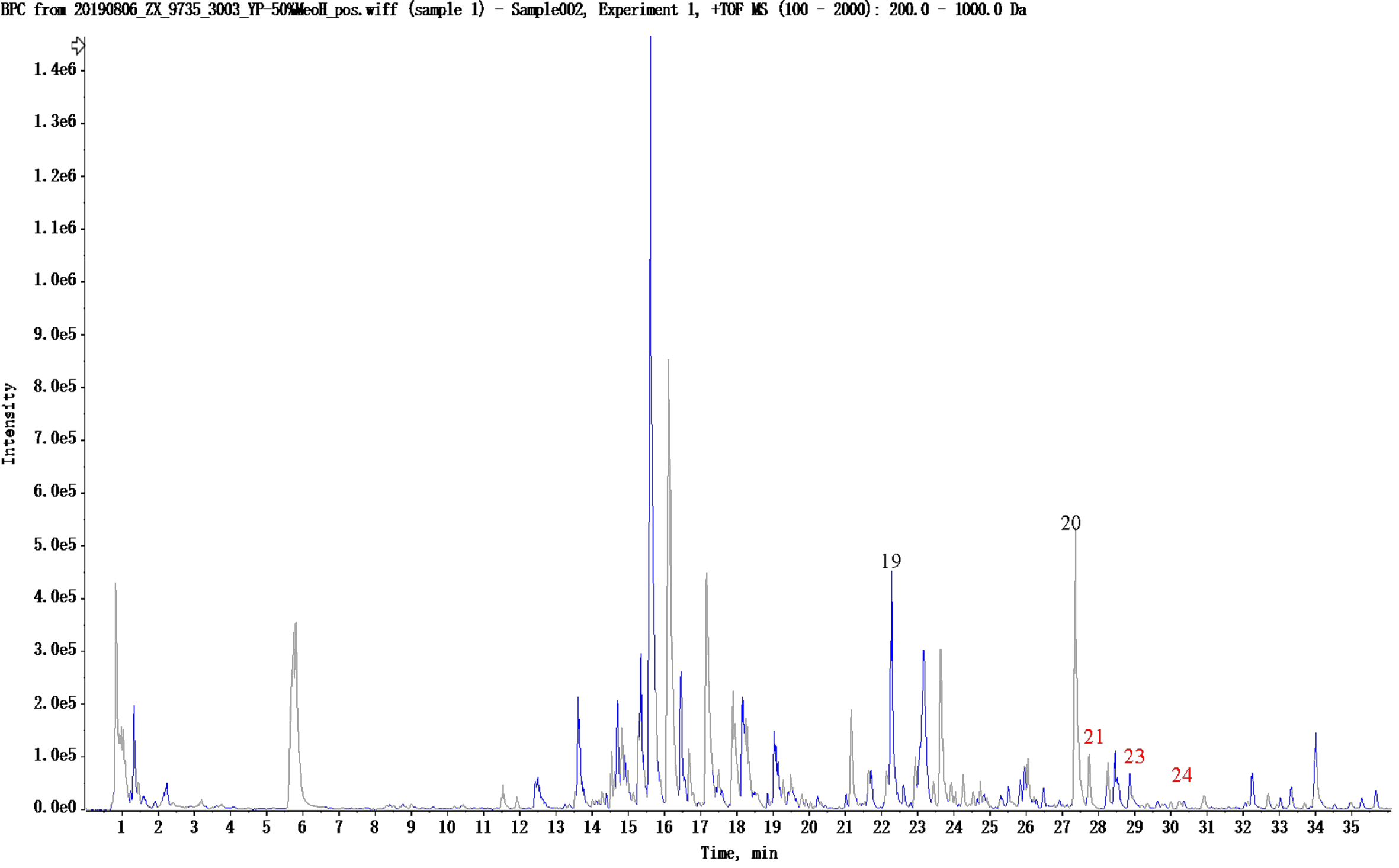

Supplement: Supplementary file 7 [file Image2.TIF]

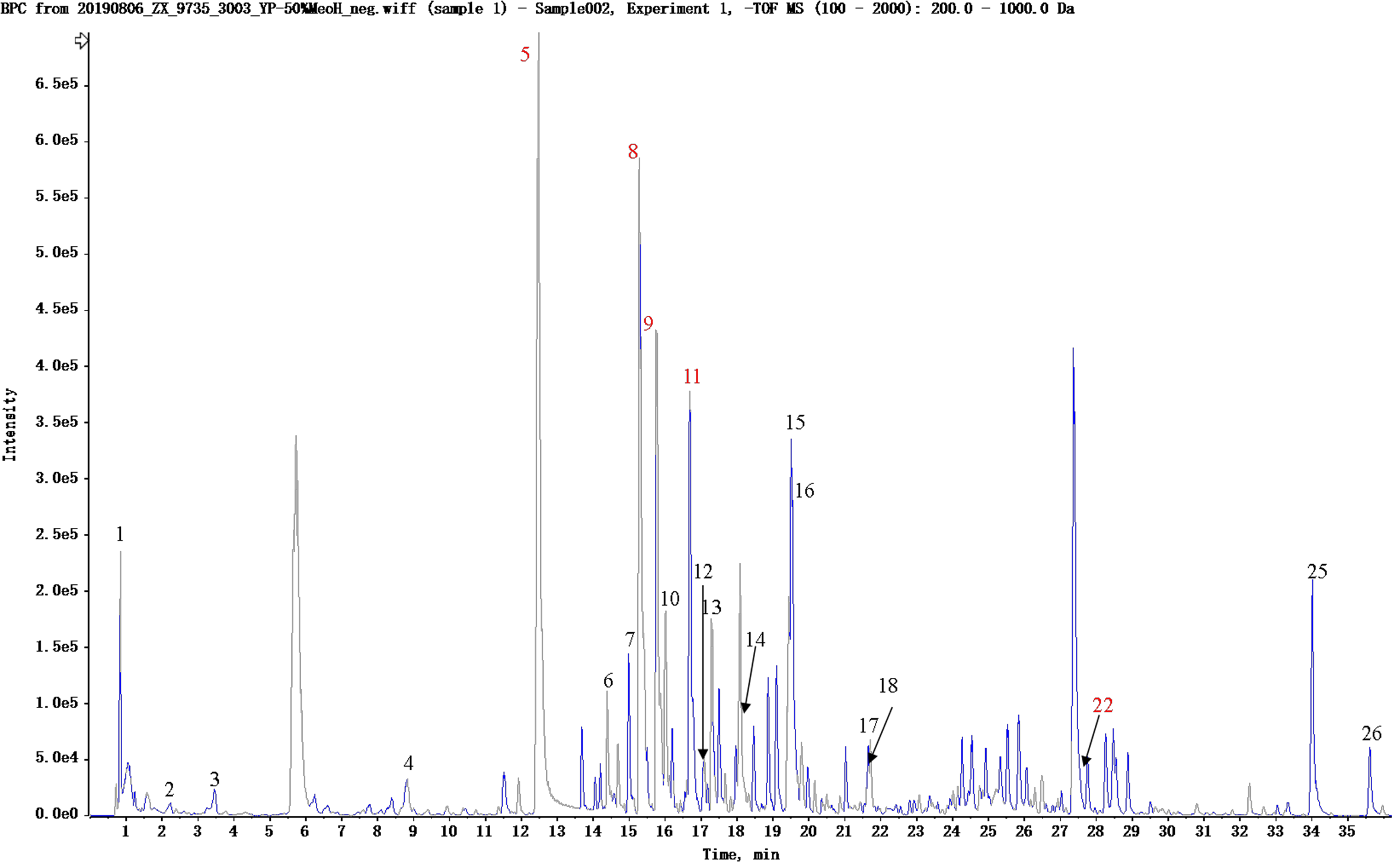

Supplement: Supplementary file 9 [file Image1.TIF]

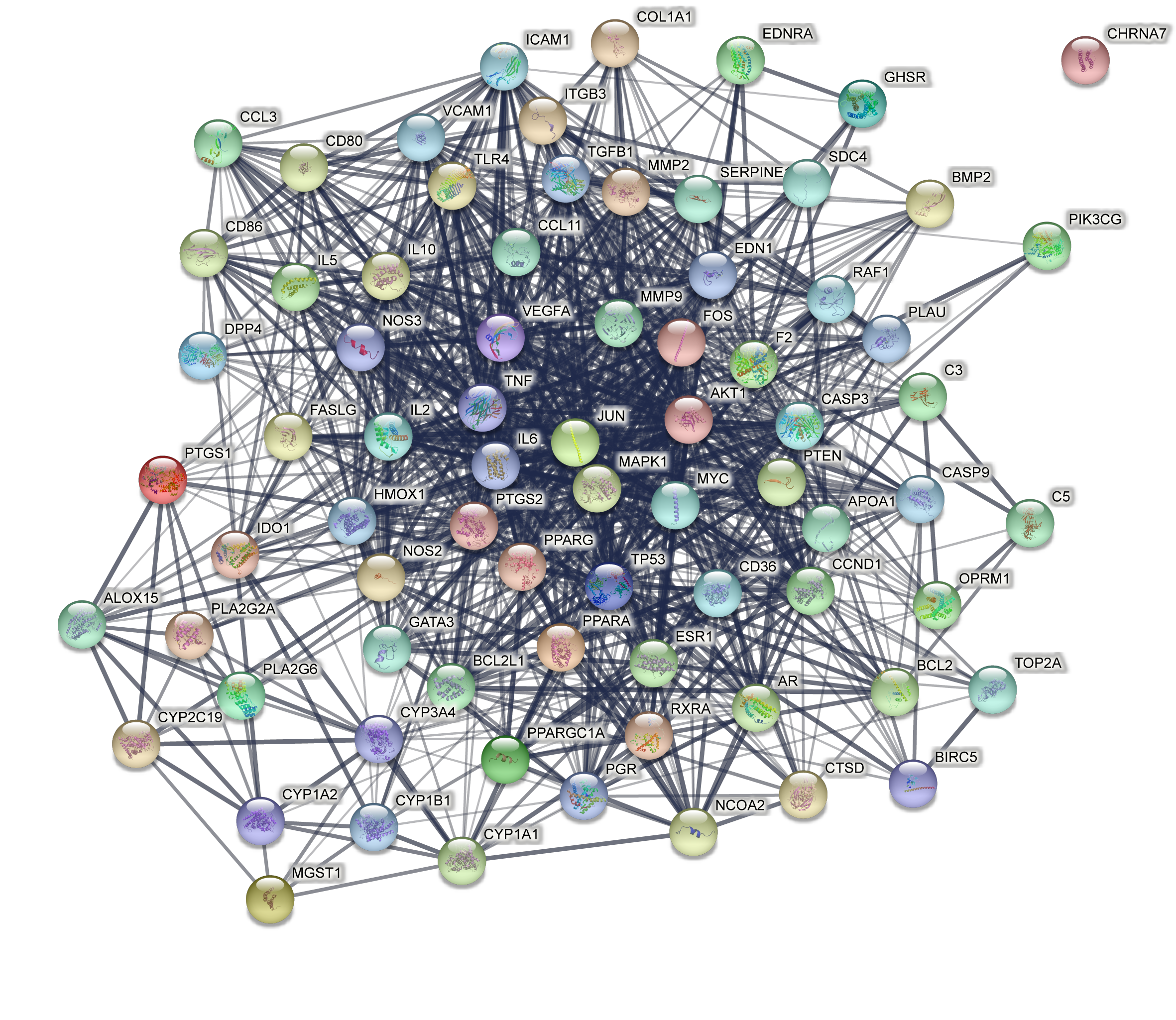

Supplement: Supplementary file 15 [file Image5.TIF]
