# Supplementary material for: Exploration of the Modulatory Property Mechanism of ELeng Capsule in the Treatment of Endometriosis Using Transcriptomics Combined With Systems Network Pharmacology
Source: Front Pharmacol. 2021 Jun 18;12:674874. doi: 10.3389/fphar.2021.674874 (PMC8249582; doi:10.3389/fphar.2021.674874)
Supplement: Supplementary file 2 [file Table1.DOCX]

**Table S1: The validated information of major plants/herbs, including location, used part,famliy,genus and medical plant references.**

| TCM name | Species Name**/Scientific Name** | Botanical Documentation | Location | Used part | Family | Genus | Medical plant reference |
| --- | --- | --- | --- | --- | --- | --- | --- |
| Ezhu | 1.*Curcuma phaeocaulis* Valeton  [Zingiberaceae]  2.*Curcuma zedoaria (Christm.) Roscoe* [Zingiberaceae] | 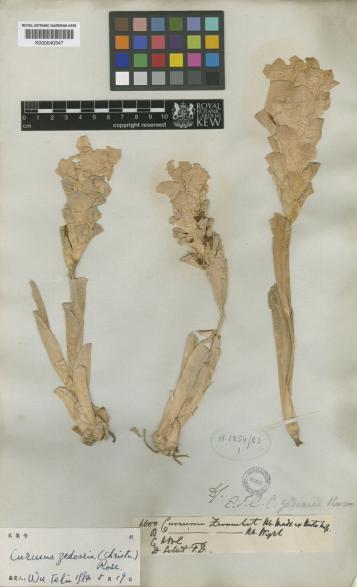 | 1.Native to:  China South-Central, Jawa, Vietnam  Introduced into:  China Southeast  2.Assam, Bangladesh, East Himalaya | Dried rhizome | Zingiberaceae Martinov | *[Curcuma L.](http://plantsoftheworldonline.org/taxon/urn:lsid:ipni.org:names:331178-2)* | The International Plant Names Index and World Checklist of Selected Plant Families 2020.  Pharmacopoeia of China (2015) .  Govaerts, R. (1999). World Checklist of Seed Plants 3(1, 2a & 2b): 1-1532. MIM, Deurne. |
| Chi Shao/Bai shao | *Paeonia lactiflora* Pall. | *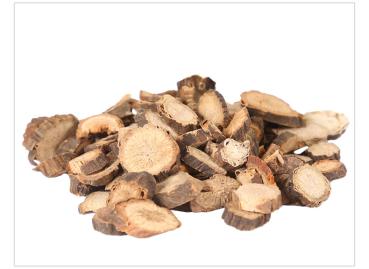* | Amur, China North-Central, China South-Central, China Southeast, Chita, Inner Mongolia, Khabarovsk, Manchuria, Mongolia, New York, Primorye | Dried root | Paeoniaceae Raf. | *Paeonia* L. | Pharmacopoeia of China (2015) .  The International Plant Names Index and World Checklist of Selected Plant Families 2020.  Paeonia albiflora Pall. Fl. Ross. 1(2): 92 (1789). |
| Danggui | *Angelica sinensis var. wilsonii (H.Wolff) Z.H.Pan & M.F.Watson*  *Angelica sinensis (Oliv.)[Diels](http://www.theplantlist.org/tpl1.1/record/kew-2639272)* | *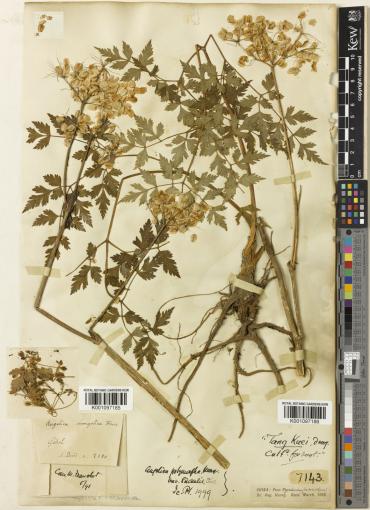* | China North-Central, China South-Central, Vietnam | Dried root | *[Apiaceae](http://plantsoftheworldonline.org/taxon/urn:lsid:ipni.org:names:30000180-2)*[Lindl.](http://plantsoftheworldonline.org/taxon/urn:lsid:ipni.org:names:30000180-2) | *[Angelica](http://plantsoftheworldonline.org/taxon/urn:lsid:ipni.org:names:39657-1)*[L.](http://plantsoftheworldonline.org/taxon/urn:lsid:ipni.org:names:39657-1) | [Chinese Pharmacopoeia Commission (ed.) (2015)](http://www.chp.org.cn/cms/home/).  The International Plant Names Index and World Checklist of Selected Plant Families 2020.  Angelica polymorpha var. sinensis Oliv. Hooker's Icon. Pl. 20: t. 1999 (1891). |
| Sanleng | *Sparganium stoloniferum* (Buch.-Ham. ex Graebn.) Buch.-Ham. ex Juz.  [Typhaceae] | 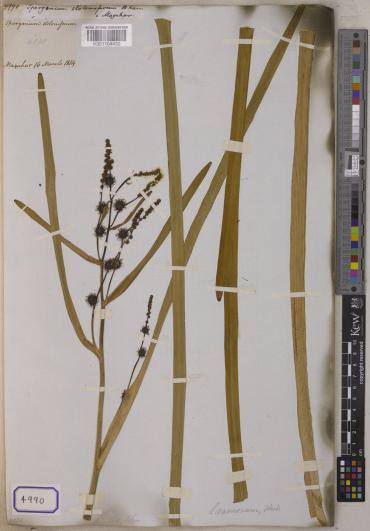 | Temp. Asia to Himalaya | Dried rhizome | *Typhaceae* Juss. | *[Sparganium](http://plantsoftheworldonline.org/taxon/urn:lsid:ipni.org:names:39598-1)*[L.](http://plantsoftheworldonline.org/taxon/urn:lsid:ipni.org:names:39598-1) | Pharmacopoeia of China (2010).  The International Plant Names Index and World Checklist of Selected Plant Families 2020.  *Sparganium erectum* subsp. stoloniferum (Buch.-Ham. ex Graebn.) H.Hara J. Jap. Bot. 51: 228 (1976). |
|  |  |  |  |  |  |  |  |
| Danshen | *Salvia miltiorrhiza*Bunge   [Lamiaceae] | 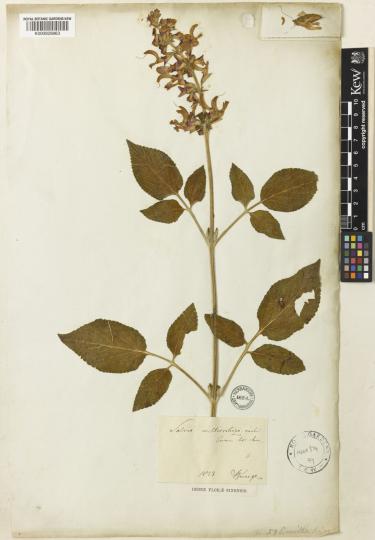 | Native to:  China North-Central, China South-Central, China Southeast, Vietnam  Introduced into:  Korea | Dried root and rhizome | [Lamiaceae Martinov](http://plantsoftheworldonline.org/taxon/urn:lsid:ipni.org:names:30000097-2) | [Salvia L.](http://plantsoftheworldonline.org/taxon/urn:lsid:ipni.org:names:30000096-2) | [Chinese Pharmacopoeia Commission (ed.) (2015)](http://www.chp.org.cn/cms/home/).  Govaerts, R. (2003). World Checklist of Selected Plant Families Database in ACCESS: 1-216203. |
| Zhike | *Citrus aurantium L.* | 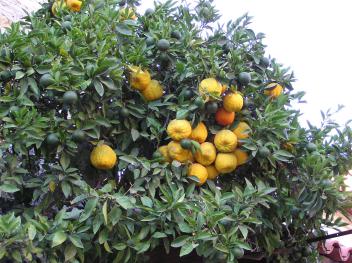 | *its native range is Artificial hybrid* | *Fruits* | [Rutaceae Juss.](http://plantsoftheworldonline.org/taxon/urn:lsid:ipni.org:names:30001492-2) | *genus [Citrus](http://www.theplantlist.org/1.1/browse/A/Rutaceae/Citrus/)* | U.S. FDA Substance Registration System (2016) |

**The validated information of plants, including TCM names,species names**/scientific names,**botanical documentation, location, used part (used part of medicinal species) and family and genus.**
