# Supplementary material for: Exploration of the Modulatory Property Mechanism of ELeng Capsule in the Treatment of Endometriosis Using Transcriptomics Combined With Systems Network Pharmacology
Source: Front Pharmacol. 2021 Jun 18;12:674874. doi: 10.3389/fphar.2021.674874 (PMC8249582; doi:10.3389/fphar.2021.674874)
Supplement: Supplementary file 3 [file Table8.DOCX]

**Table S8:The expression of VEGFA,VEGFB in serum（pg/m1，****$\overline{x}$±s，n=8）**

| Group | n | VEGFA | VEGFB |
| --- | --- | --- | --- |
| Control | 8 | 34.838±1.403 ^*^ | 18.66±23.03 |
| Model | 8 | 38.866±2.706 | 31.971±26.300 |
| ELC high group | 8 | 35.345±4.205 ^**^ | 21.785±23.870 |
| ELC middle group | 8 | 36.777±2.745 | 38.261±20.758 |
| ELC low group | 8 | 35.024±2.662^***^ | 25.168±22.890 |
| *F* |  | 2.742 | 0.674 |
| *P* |  | 0.044 | 0.614 |
