# Supplementary material for: Exploration of the Modulatory Property Mechanism of ELeng Capsule in the Treatment of Endometriosis Using Transcriptomics Combined With Systems Network Pharmacology
Source: Front Pharmacol. 2021 Jun 18;12:674874. doi: 10.3389/fphar.2021.674874 (PMC8249582; doi:10.3389/fphar.2021.674874)
Supplement: Supplementary file 6 [file Table3.DOC]

**Table S3 The result of compounds identification in ELeng Capsules**

| No | Time（min） | Additive ion | M/Z Actual value | M/Z Theoretical value | ppm | Molecular formula | Name | MS/MS spcetra | Chinese herbs |
| --- | --- | --- | --- | --- | --- | --- | --- | --- | --- |
| 1 | 0.83 | [M-H]- | 341.1089 | 341.1089 | -0.1 | C12H22O11 | Sucrose | 341.1095,179.0560,89.0244 | *Citrus aurantium L.*  *,Angelica sinensis (Oliv.)Diels* |
| 2 | 2.05 | [M-H]- | 169.015 | 169.0142 | 4.5 | C7H6O5 | Gallic acid | 169.0172,125.0240,79.0196 | *Sparganium stoloniferum* |
| 3 | 3.45 | [M-H]- | 197.0456 | 197.0455 | 0.3 | C9H10O5 | Danshensu | 179.0352,135.0459,123.0448,72.9917 | *Salvia miltiorrhiza Bunge* |
| 4 | 8.8 | [M-H]- | 495.15 | 495.1508 | -1.6 | C23H28O12 | Oxypaeoniflora | 495.1503,465.1395,165.0558,137.0245,93.0340 | *Paeonia lactiflora Pall.* |
| 5 | 12.47 | [M+FA]- | 525.1618 | 525.1614 | 0.8 | C23H28O11 | Paeoniflorin | 449.1444,327.1075,165.0551,121.0283 | *Paeonia lactiflora Pall.* |
| 6 | 14.4 | [M-H]- | 595.1693 | 595.1704 | -1.8 | C27H32O15 | Neoeriocitrin | 595.1663,459.1138,339.0707,287.0549,235.0235,151.0029,135.0448 | *Citrus aurantium L.* |
| 7 | 14.99 | [M-H]- | 579.1733 | 579.1719 | 2.4 | C27H32O14 | Narirutin | 579.1745,271.0605,151.0034 | *Citrus aurantium L.* |
| 8 | 15.29 | [M-H]- | 579.1732 | 580.54 | 2.2 | C27H32O14 | Naringin | 549.1717,459.1154,313.0710,271.0594,177.0183,151.0027,119.0492 | *Citrus aurantium L.* |
| 9 | 15.77 | [M-H]- | 609.182 | 609.1825 | -0.8 | C28H34O15 | Neohesperidin | 609.1813,489.1405,343.0813,301.0694,286.0455,242.0564,164.0102 | *Citrus aurantium L.* |
| 10 | 16.01 | [M-H]- | 359.0772 | 359.0772 | -0.1 | C18H16O8 | Rosmarinic acid | 197.0457,179.0349,161.0244,135.0450,133.0293,123.042,72.9931 | *Salvia miltiorrhiza Bunge* |
| 11 | 16.69 | [M-H]- | 717.1475 | 717.1461 | 1.9 | C36H30O16 | Salvianolic acid B | 717.1460,519.0935,339.0507,321.0393,295.0610,279.0296,185.0246 | *Salvia miltiorrhiza Bunge* |
| 12 | 17.09 | [M-H]- | 315.1605 | 315.1602 | 1 | C19H24O4 | 1,7-bis(4-hydroxyphenyl)-3,5-heptanediol | 163.0748,149.0607,121.0656,106.0412,93.0340 | *Curcuma phaeocaulis* Valeton |
| 13 | 17.3 | [M-H]- | 493.1135 | 493.114 | -1.1 | C26H22O10 | Salvianolic acid | 493.1152,313.0720,295.0612,185.0246,109.0290 | *Salvia miltiorrhiza Bunge* |
| 14 | 18.17 | [M-H]- | 267.031 | 267.0299 | 4.1 | C15H8O5 | Sparstolonin B | 267.0308,182.0425,167.0593,154.9976 | *Sparganium stoloniferum* |
| 15 | 19.45 | [M-H]- | 301.0715 | 301.0718 | -0.9 | C16H14O6 | Hesperetin | 301.0717,164.0118,151.0040,136.0169 | *Citrus aurantium L.* |
| 16 | 19.46 | [M-H]- | 329.2337 | 329.2333 | 1.1 | C18H34O5 | sanleng acid | 309.2328,229.1444,211.1342,193.1230,183.1389,171.1028,139.1129,127.1131,99.0816 | *Sparganium stoloniferum* |
| 17 | 21.65 | [M-H]- | 407.2816 | 407.2803 | 3.2 | C24H40O5 | Cholic acid | 407.2807,343.2621 | Unkown |
| 18 | 21.72 | [M+FA]- | 515.1941 | 515.1923 | 3.5 | C26H30O8 | Limonin | 515.1942,469.1885,278.1320,229.1237 | *Citrus aurantium L.* |
| 19 | 22.07 | [M+H]+ | 237.1845 | 237.1849 | -1.7 | C15H24O2 | Curdione | 163.1117,137.0957,121.1012,107.0853,91.0540,79.536,79. 0536,67.0536 | *Curcuma phaeocaulis* Valeton |
| 20 | 27.38 | [M+H]+ | 279.2323 | 279.2319 | 1.6 | C18H30O2 | Linolenic acid | 295.2279,277.2176,195.1396,171.1031 | *Salvia miltiorrhiza Bunge* |
| 21 | 27.71 | [M+H]+ | 297.1483 | 297.1485 | -0.7 | C19H20O3 | Cryptotanshinone | 297.1479,179.1380,25.0930,251.1423,249.0908,139.0710,221.0961,193.1015,178.0770 | *Salvia miltiorrhiza Bunge* |
| 22 | 27.71 | [M-H]- | 277.0863 | 277.0859 | 1.4 | C18H12O3 | Tanshinone I | 277.0854,249.0903,234.193.0998,178.0770 | *Slavia miltiorrhiza* |
| 23 | 28.94 | [M+H]+ | 219.1742 | 219.1743 | -0.6 | C15H22O | (E,E)-Germacra-3,7(11),9-trien-6-one | 159.1155,129.0692,105.0689,95.0847,91.0536,79.0532,67.0532,55.0535 | *Curcuma phaeocaulis* |
| 24 | 30.19 | [M+H]+ | 295.1335 | 293.1183 | 2.1 | C19H18O3 | Tanshinone IIA | 295.1482,277.1377,213.0663,163.1134,151.1120,107.0879,93.0699 | *Slavia miltiorrhiza* |
| 25 | 34.01 | [M-H]- | 279.2337 | 279.233 | 2.7 | C18H32O2 | Linoleic acid | 279.2334 | *Slavia miltiorrhiza* |
| 26 | 35.61 | [M-H]- | 255.2331 | 255.233 | 0.6 | C16H32O2 | Palmitic acid | 255.2321 | *Citrus aurantium,Slavia miltiorrhiza*  *,Sparganium stoloniferum* |
