# Supplementary material for: Exploration of the Modulatory Property Mechanism of ELeng Capsule in the Treatment of Endometriosis Using Transcriptomics Combined With Systems Network Pharmacology
Source: Front Pharmacol. 2021 Jun 18;12:674874. doi: 10.3389/fphar.2021.674874 (PMC8249582; doi:10.3389/fphar.2021.674874)
Supplement: Supplementary file 8 [file Table7.DOCX]

| Group | n | Volumes 1(V1)  （x±s）(mm^3^) | Volumes 2(V2)（x±s）(mm^3^) | Volumes change (V1-V2)  （x±s）(mm^3^) |
| --- | --- | --- | --- | --- |
| Model group | 10 | 21.38±11.03 | 26.01±20.90 | -4.63±15.15 |
| ELC high group | 10 | 24.35±20.66 | 9.99±10.21 | 14.36±21.019 |
| ELC middle group | 10 | 36.95±19.87 | 15.56±8.82 | 21.39±20.19^*^ |
| ELC low group | 10 | 24.01±19.87 | 22.65±23.97 | 1.36±6.38 |

**Table S7:The comparison of lesion volume between endometriosis model rats before and after treatment (mm^3^)**

Volumes 1(V1):before treatment,Volumes 2(V2):after treatment.

The volume comparison after ELC treatment showed that ELC could reduce the volume of lesions in endometriosis model rats.Data are presented as mean ± SD(n=10), *P*<0.05 .**P*=0.028＜0.05.
