# Supplementary material for: Exploration of the Modulatory Property Mechanism of ELeng Capsule in the Treatment of Endometriosis Using Transcriptomics Combined With Systems Network Pharmacology
Source: Front Pharmacol. 2021 Jun 18;12:674874. doi: 10.3389/fphar.2021.674874 (PMC8249582; doi:10.3389/fphar.2021.674874)
Supplement: Supplementary file 10 [file Table9.DOCX]

**Table S9:The expression of DEGs in Model_ecto vs ELC_ecto group by qPCR**

| genes | n | Model_ecto group | ELC_ecto group | *t* | *P* |
| --- | --- | --- | --- | --- | --- |
| Smyd1 | 7 | 4.502±4.746 | 0.417±0.697 | 2.253 | 0.0438 |
| Six1 | 7 | 2.164 ± 0.8954 | 0.2634 ± 0.1014 | 2.109 | 0.0566 |
| Cacna1s | 7 | 8.574 ± 3.577 | 0.6445 ± 0.443 | 2.2 | 0.0481 |
| Eef1a2 | 7 | 5.267 ± 2.107 | 0.486 ± 0.3355 | 2.241 | 0.0447 |
| Myog | 7 | 3.436±3.473 | 0.383±0.678 | 1.057 | 0.3155 |
| Ryr1 | 7 | 9.368 ± 4.519 | 0.5591 ± 0.3412 | 1.944 | 0.0758 |
| Actn2 | 7 | 3.196 ± 1.324 | 0.4252 ± 0.237 | 2.06 | 0.0618 |
| Mapk12 | 6 | 1.423 ± 0.5089 | 0.7529 ± 0.2277 | 1.201 | 0.2573 |
| Myod1 | 6 | 6.339 ± 3.541 | 2.148 ± 1.786 | 1.057 | 0.3155 |
| Myh4 | 6 | 17.67 ± 9.731 | 12.14 ± 9.815 | 0.4 | 0.6976 |
