# Supplementary material for: Exploration of the Modulatory Property Mechanism of ELeng Capsule in the Treatment of Endometriosis Using Transcriptomics Combined With Systems Network Pharmacology
Source: Front Pharmacol. 2021 Jun 18;12:674874. doi: 10.3389/fphar.2021.674874 (PMC8249582; doi:10.3389/fphar.2021.674874)
Supplement: Supplementary file 12 [file Table2.DOCX]

### Table 2 Chromatographic conditions

1.Flow phase gradient

| Time (min) | Flow Rate (mL/min) | %B |
| --- | --- | --- |
| 0 | 0.3 | 5 |
| 3 | 0.3 | 5 |
| 11 | 0.3 | 14 |
| 36 | 0.3 | 95 |
| 38 | 0.3 | 95 |
| 38.1 | 0.3 | 5 |
| 41 | 0.3 | 5 |

2.MS parameter

| parameter | value |
| --- | --- |
| TOF mass range | 50～1500 |
| Ion Source Gas 1 | 50 |
| Ion Source Gas 2 | 50 |
| Curtain Gas | 35 |
| Ion Spray Voltage Floating (kV) | -4500/5000 |
| Ion Source Temperature (°C) | 500 |
| Declustering Potential | 100 |
| Collision Energy | 10 |

3.MS/MS parameter

| parameter | value |
| --- | --- |
| Declustering Potential | 100 |
| Collision Energy | ±40 |
| Collision Energy Spread | 20 |
| Ion Release Delay | 30 |
| Ion Release Width | 15 |
