# Supplementary material for: Exploration of the Modulatory Property Mechanism of ELeng Capsule in the Treatment of Endometriosis Using Transcriptomics Combined With Systems Network Pharmacology
Source: Front Pharmacol. 2021 Jun 18;12:674874. doi: 10.3389/fphar.2021.674874 (PMC8249582; doi:10.3389/fphar.2021.674874)
Supplement: Supplementary file 13 [file Table4.DOCX]

|  | Time（min） | Compounds | Molecular formula | Actual value | Relative average content（%） |
| --- | --- | --- | --- | --- | --- |
| 1 | 8.647 | Eucalyptol | C_10_H_18_O | 154 | 0.60 |
| 2 | 10.589 | D-Camphor | C_10_H_16_O | 152 | 1.33 |
| 3 | 10.773 | Isoborneol | C_10_H_18_O | 154 | 0.96 |
| 4 | 10.903 | L(-)-Borneol | C_10_H_18_O | 154 | 0.5 |
| 5 | 11.270 | α-Terpineol | C_10_H_18_O | 154 | 0.2 |
| 6 | 14.344 | b-Elemen | C_15_H_24_ | 204 | 3.69 |
| 7 | 15.093 | γ-Elemen | C_15_H_24_ | 204 | 0.73 |
| 8 | 15.591 | α-Humulene | C_15_H_24_ | 204 | 0.34 |
| 9 | 20.683 | Germacra | C_15_H_22_O | 218 | 10.04 |
| 10 | 21.334 | Curcumenol | C_15_H_22_O_2_ | 234 | 37.22 |
| 11 | 22.871 | b-Cyclocostunolide | C_15_H_20_O_2_ | 232 | 4.16 |
| 12 | 24.025 | Curcumenone | C_15_H_22_O_2_ | 234 | 5.23 |
| 13 | 27.344 | Zederone | C_15_H_18_O_3_ | 246 | 1.34 |
| 14 | 28.124 | ent-Kaurene | C_20_H_32_ | 272 | 8.38 |

**Table S4** **The compounds of ELeng Capsule based on GS-MS**
